# IFITM3 regulates fibrinogen endocytosis and platelet reactivity in nonviral sepsis

**DOI:** 10.1172/JCI153014

**Published:** 2022-12-01

**Authors:** Robert A. Campbell, Bhanu Kanth Manne, Meenakshi Banerjee, Elizabeth A. Middleton, Abigail Ajanel, Hansjorg Schwertz, Frederik Denorme, Chris Stubben, Emilie Montenont, Samantha Saperstein, Lauren Page, Neal D. Tolley, Diana L. Lim, Samuel M. Brown, Colin K. Grissom, Douglas W. Sborov, Anandi Krishnan, Matthew T. Rondina

**Affiliations:** 1 University of Utah Molecular Medicine Program, Salt Lake City, Utah, USA.; 2 Department of Internal Medicine,; 3 Department of Pathology, and; 4 Rocky Mountain Center for Occupational and Environmental Health, University of Utah, Salt Lake City, Utah, USA.; 5 Occupational Medicine, Billings Clinic Bozeman, Bozeman, Montana, USA.; 6 Bioinformatics Shared Resource, Huntsman Cancer Institute, University of Utah, Salt Lake City, Utah, USA.; 7 Division of Pulmonary and Critical Medicine, Department of Medicine, Intermountain Medical Center, Murray, Utah, USA.; 8 Huntsman Cancer Institute, University of Utah, Salt Lake City, Utah, USA.; 9 Stanford Cancer Institute, Stanford University School of Medicine, Stanford, California, USA.; 10 Department of Pathology, Stanford University, Stanford, California, USA.; 11 George E. Wahlen Department of Veterans Affairs Medical Center, Department of Internal Medicine, and Geriatric Research, Education, and Clinical Center, Salt Lake City, Utah, USA.

**Keywords:** Hematology, Inflammation, Bacterial infections, Platelets, Thrombosis

## Abstract

Platelets and megakaryocytes are critical players in immune responses. Recent reports suggest infection and inflammation alter the megakaryocyte and platelet transcriptome to induce altered platelet reactivity. We determined whether nonviral sepsis induces differential platelet gene expression and reactivity. Nonviral sepsis upregulated IFN-induced transmembrane protein 3 (IFITM3), an IFN-responsive gene that restricts viral replication. As IFITM3 has been linked to clathrin-mediated endocytosis, we determined whether IFITM3 promoted endocytosis of α-granule proteins. IFN stimulation enhanced fibrinogen endocytosis in megakaryocytes and platelets from *Ifitm^+/+^* mice, but not *Ifitm^–/–^* mice. IFITM3 overexpression or deletion in megakaryocytes demonstrated IFITM3 was necessary and sufficient to regulate fibrinogen endocytosis. Mechanistically, IFITM3 interacted with clathrin and α_IIb_ and altered their plasma membrane localization into lipid rafts. In vivo IFN administration increased fibrinogen endocytosis, platelet reactivity, and thrombosis in an IFITM-dependent manner. In contrast, *Ifitm^–/–^* mice were completely rescued from IFN-induced platelet hyperreactivity and thrombosis. During murine sepsis, platelets from *Ifitm^+/+^* mice demonstrated increased fibrinogen content and platelet reactivity, which was dependent on IFN-α and IFITMs. Platelets from patients with nonviral sepsis had increases in platelet IFITM3 expression, fibrinogen content, and hyperreactivity. These data identify IFITM3 as a regulator of platelet endocytosis, hyperreactivity, and thrombosis during inflammatory stress.

## Graphical abstract

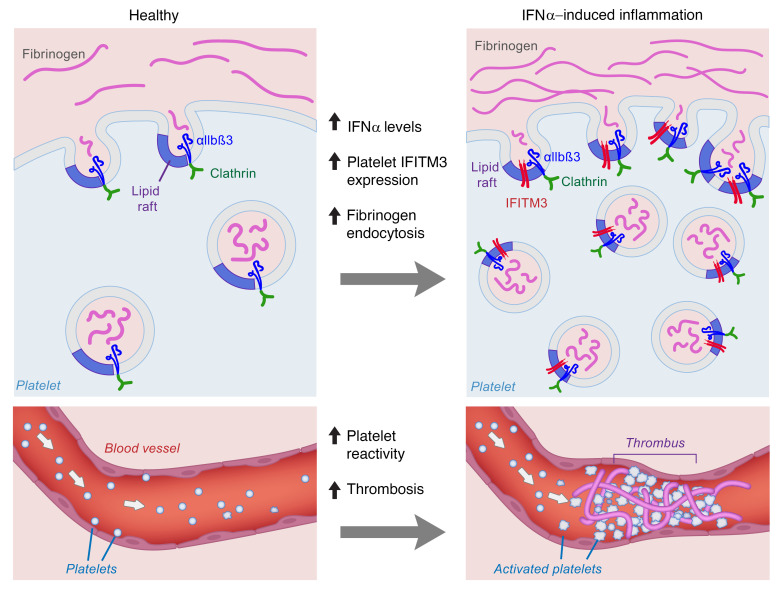

## Introduction

Sepsis affects approximately 50 million individuals worldwide each year and remains one of the leading causes of infection-related hospitalizations and death (1, 2). The most common causes of sepsis are Gram-positive bacteria, such as *Staphylococcus aureus* and *Streptococcus pneumoniae*, as well as Gram-negative bacteria, including *Escherichia coli* (3, 4*)*. Infections progress systemically when the host immune response system fails to limit the spread of the pathogen (5). While the spread of infection contributes to the development and severity of sepsis, the host’s dysregulated inflammatory response is another driver of disease progression (6). Alterations in the host’s immune system and organ homeostasis contribute to development of sepsis. In addition, dysregulated coagulation, including both thrombocytopenia and, independently, platelet hyperreactivity, are associated with increased sepsis-related morbidity and mortality (7, 8). This includes the development of micro- and macrovascular thrombosis, which further contributes to poor outcomes (8, 9). Sepsis survivors also have an increased longer-term risk of thromboembolic events, including venous thromboembolism (10, 11).

Platelets are small (2–4 μm), anucleate, cytoplasmic fragments released in the bloodstream from megakaryocytes located in the bone marrow (12). While platelets are key regulators of hemostasis and vascular integrity (13), they are centrally positioned in the vasculature to participate in immunosurveillance (14). Megakaryocytes and platelets possess a broad array of receptors, including TLRs, and interact with other immune cells, including monocytes, neutrophils, and lymphocytes (14). Through these receptors and associated pathways, platelets can sense and help clear invading pathogens and bacteria (15–19). While megakaryocytes and platelets play critical roles in the immune response, an accumulating body of evidence indicates that infection and inflammation can alter the megakaryocyte and platelet transcriptome and proteome, resulting in the release of platelets into the circulation with altered functions (19–22).

The IFN-induced transmembrane (IFITM) proteins comprise a family of IFN-induced antiviral restriction factors with constitutive expression in many cells (23, 24). Located on chromosome 11 in humans and chromosome 4 in mice, these innate immune receptors are upregulated by type I and type II IFNs, which are increased in sepsis (23, 24). IFITMs are known to play critical roles in restricting viral replication. We recently demonstrated that IFITM3 increases in megakaryocytes and platelets during dengue and COVID-19 infection and limits dengue virus entry into the cytoplasm of megakaryocytes, resulting in reduced viral replication (19, 21). Mechanistically, IFITM3 regulates viral infections in part through modulating endocytosis. IFITM3 allows for endocytosis of viral particles, but prevents engulfed viruses from fusing with late endosomes, thereby blocking viral replication (25–29). IFITM3 possesses a YxxΦ-sorting motif that enables itself to interact with AP-2, a protein important in clathrin-mediated endocytosis (CME) (27, 28). In platelets, CME regulates the cellular uptake of procoagulant, inflammatory, and immune proteins, thereby mediating thromboinflammatory responses (30). While the antiviral role of IFITM3 is well established, whether IFITM3 orchestrates CME in megakaryocytes and platelets and whether this results in prothrombotic responses in nonviral sepsis has not previously been examined.

Here, we demonstrate IFITM3 is upregulated in platelets isolated from acutely infected patients with nonviral sepsis. Increased transcription and translation of IFITM3 was dependent on IFN activation of STAT1 and mTOR, respectively. While IFITM3 is known to regulate endocytosis in classical immune cells, we demonstrate that IFITM3 participates in CME in megakaryocytes and platelets by regulating uptake of α-granule proteins, including fibrinogen and transferrin. The increase in fibrinogen endocytosis was dependent on IFITM3 and resulted in enhanced platelet reactivity, as demonstrated by increased platelet aggregation. Furthermore, ablating IFITM3 was protective against an inflammation-dependent increase in pulmonary microvascular thrombosis. Mechanistically, IFITM3 directly interacted with and altered the localization of clathrin and the fibrinogen receptor α_IIb_ into lipid rapids in megakaryocytes and platelets. Importantly, increased expression of IFITM3 on platelets from nonviral sepsis patients was associated with increases in fibrinogen endocytosis and platelet hyperactivity as well as shifting of clathrin and α_IIb_ into platelet lipid rafts.

Thus, our findings show that during nonviral infections, IFITM3 is upregulated in human megakaryocytes and platelets by IFN-dependent activation of transcriptional and translational pathways. During sepsis and under inflammatory stress, IFITM3 alters lipid raft localization of critical platelet proteins such as clathrin and α_IIb_. Upregulation of platelet IFITM3 and increased fibrinogen CME promotes platelet hyperactivity and contributes to thrombosis. These findings highlight what we believe is a previously unrecognized role of IFITM3 in cellular biology and elucidate a therapeutic target for helping to prevent microvascular thrombosis in sepsis. As IFITM3 is ubiquitously expressed and CME regulates many cellular responses, our findings may have relevance to a broad repertoire of cells and tissues.

## Results

Clinical characteristics of healthy donors and patients with nonviral sepsis are summarized in Supplemental Table 1 (supplemental material available online with this article; https://doi.org/10.1172/JCI153014DS1). Subjects were well matched by age and sex. Septic patients tended to be obese with underlying comorbidities, including diabetes and hypertension, as is expected and common in studies of this nature. Approximately 20% of patients were mechanically ventilated, while almost half of the patients had septic shock and were on vasopressors. Patients had elevated white blood cell counts, while platelet counts were in the normal range based on reference ranges from ARUP Laboratories. While approximately 25% of sepsis patients were on antiplatelet medications (aspirin), platelet aggregation assays were only performed on patients not on aspirin. In our cohort, we observed a mortality rate of 7%.

### Nonviral sepsis induces an increase in platelet IFITM3 expression.

**Figure 1:**
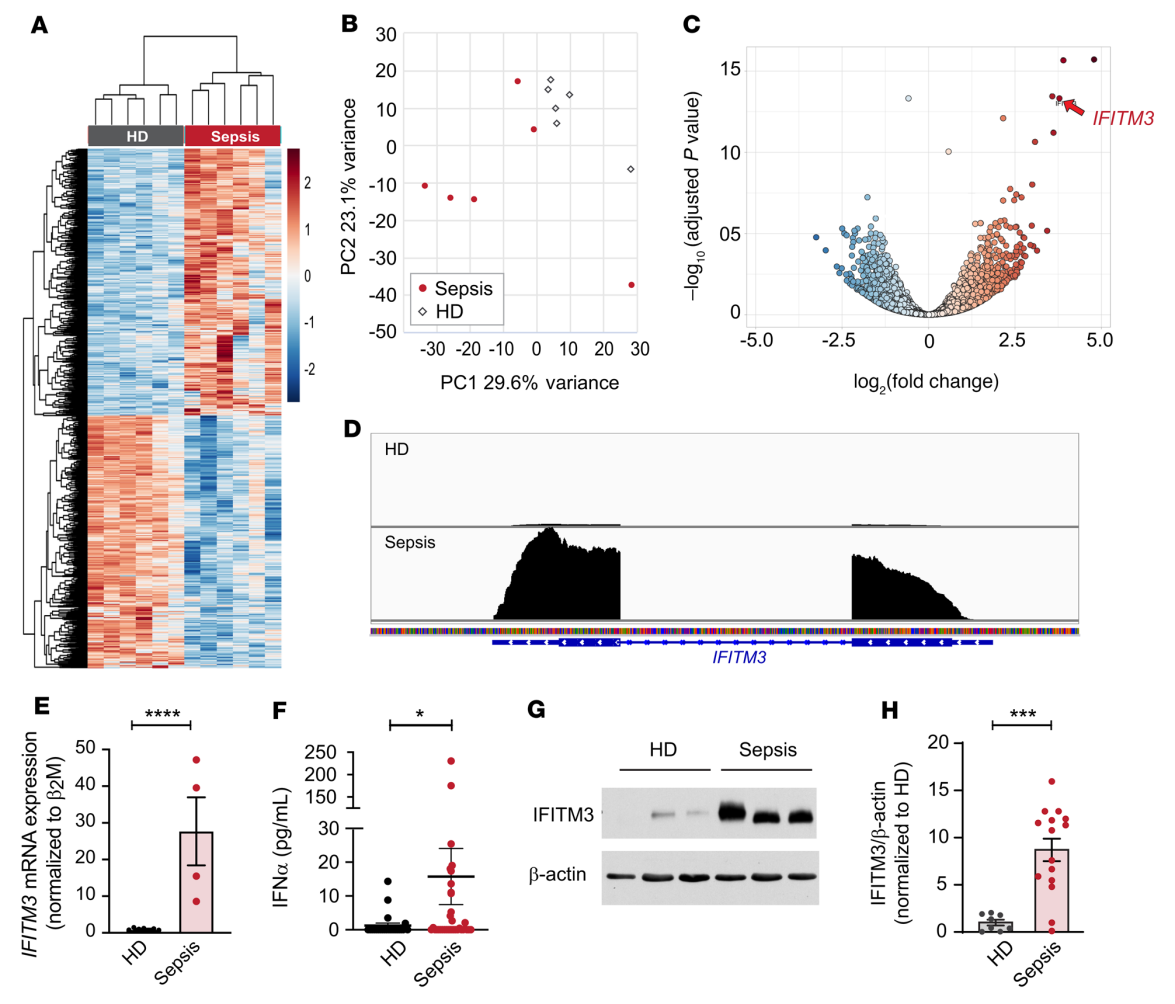
Platelet IFITM3 expression increases in nonviral sepsis. Platelets were isolated from patients with nonviral sepsis or matched healthy donors (see also Supplemental Table 2). Purified platelets were isolated as described in Methods. (**A**) Heatmap of significantly differentially expressed (adjusted *P* < 0.05) transcripts in nonviral sepsis patients (*n* = 6) and healthy donors (HD) (*n* = 6). Red shows significantly enriched transcripts, and blue shows significantly repressed transcripts. (**B**) Principal component analysis (PCA) demonstrating data from nonviral sepsis patients cluster together, while data from healthy donors cluster together. (**C**) Scatter plot with significantly enriched (red) and repressed (blue) transcripts. *IFITM3* is indicated (arrow). (**D**) Representative Integrative Genomics Viewer (IGV) browser image of *IFITM3* mRNA expression in a nonviral sepsis patient (bottom) and healthy donor (top). The vertical axis represents the relative amount of mRNA, and the horizontal axis shows the introns (thin lines) and exons (thick lines). (**E**) In an independent cohort of nonviral sepsis patients (*n* = 4), platelet IFITM3 expression increases compared with that of healthy donors (*n* = 7). ****P ≤ 0.0001, unpaired *t* test. (**F**) Whole blood was drawn from nonviral sepsis patients (*n* = 34) and healthy controls (*n* = 23), and plasma levels of IFN-α were measured. *P ≤ 0.05, Mann-Whitney *U* test. (**G** and **H**) Representative immunoblot and densitometric quantification of IFITM3 and β-actin expression in platelets isolated from healthy donors (*n* = 8) and nonviral sepsis patients (*n* = 15). ***P ≤ 0.001, unpaired *t* test.

We performed RNA-Seq on highly purified, CD45-depleted platelets from nonviral sepsis patients admitted to the Medical Intensive Care Unit at the≈University of Utah Health Sciences Center in Salt Lake City and healthy age- and sex-matched donors (Supplemental Table 2). Differential gene analyses identified 604 upregulated and 604 downregulated transcripts at a significant FDR (*P* < 0.05, Figure 1A). In a principal component analysis, nonviral septic patients generally clustered together (Figure 1B). We observed that *IFITM3* was 1 of the top 5 differentially expressed transcripts in platelets from septic patients (3.8-fold increase, *P* = 2.5 × 10^–14^; Figure 1, C and D). The expression of *IFITM1* and *IFITM2* in platelets was also significantly increased, although the changes were less pronounced, 1.25-fold (*P* = 0.02) and 2.5-fold (*P* = 2.1 × 10^–8^), respectively. Changes in *IFITM3* expression were about 35-fold higher than in *IFITM1* and 2.3-fold higher than in *IFITM2*. No other IFITMs were significantly upregulated in nonviral sepsis patients (Supplemental Table 3). In addition, we observed a significant increase in heparanase (*HPSE*) (3.1-fold increase) (31), galectin 3 binding protein (*LGALS3BP*) (3.0-fold increase) (32), and selenium binding protein 1 (*SELENBP1*) (3.0-fold increase) (33), which have been observed to be increased systemically during infection and inflammation, but have not been well studied in platelets. In a separate, independent cohort of septic patients, real-time PCR validated the significant increase in platelet *IFITM3* mRNA expression (Figure 1E).

Because IFITMs are IFN-sensitive genes, we measured systemic IFN levels in our cohort of patients with nonviral sepsis. Plasma levels of IFN-α, a type I IFN, were significantly increased in sepsis patients compared with matched healthy subjects (Figure 1F). Intriguingly, plasma levels of IFN-γ, a type II IFN, were not significantly different in sepsis patients compared with healthy donors (Supplemental Figure 1). As the transcript for IFITM3 was upregulated in platelets during sepsis (Figure 1, C and D), we next determined whether IFITM3 protein expression in platelets was coordinately increased. IFITM3 protein was generally not detected in platelets isolated from healthy donors (Figure 1, G and H). In contrast, IFITM3 protein was highly expressed and significantly upregulated in platelets isolated from the majority of sepsis patients (Figure 1, G and H). Furthermore, ribosomal footprinting analysis examining actively translated transcripts in platelets from septic patients and matched, healthy donors demonstrated *IFITM3* was one of the most differentially, translationally upregulated RNAs and that *IFITM3* RNA had increased ribosomal-protected regions in septic patients (3.25-fold increase, *P* = 0.0005) (20). Interestingly, IFN-α levels modestly and negatively correlated with IFITM3 expression in nonviral sepsis patients, suggesting that in our patient cohort, systemic IFN-α levels may have been high before enrollment and were decreasing at the time we collected blood samples (Supplemental Figure 2). Taken together, these data indicate that in health, IFITM3 is transcriptionally and translationally silent in platelets and that during nonviral sepsis, IFITM3 mRNA and protein are actively made.

### IFNs regulate IFITM3 expression through STAT1 and mTOR.

**Figure 2:**
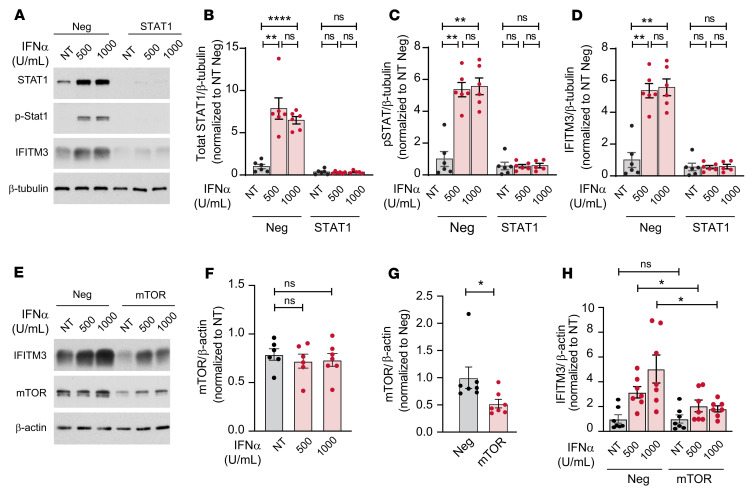
IFN-induced IFITM3 expression is dependent on STAT1 and mTOR pathways. (**A**–**D**) CD34^+^-derived cells were transfected on day 5 of culture with negative (Neg) control or STAT1 crRNA. Megakaryocytes were then stimulated with IFN-α (500 or 1000 U/mL, final) or no treatment (NT) on day 13. After 24 hours, megakaryocytes were lysed and probed to examine total STAT1 (**B**), p-STAT1 (**C**), and IFITM3 expression by immunoblot (**D**). β-Tubulin was used as a loading control. Representative blots of total STAT1, p-STAT1, and IFITM3 expression are shown (**A**–**D**). Statistical analysis used was a mixed effect analysis with Tukey’s multiple comparison test with values normalized to NEG NT (*n* = 5–6). **P ≤ 0.01; ****P ≤ 0.0001. (**E**–**H**) CD34^+^-derived cells were transfected on day 5 of culture with negative control or mTOR crRNA. Megakaryocytes were then stimulated with IFN-α (500 or 1000 U/mL, final) or no treatment on day 13. After 24 hours, megakaryocytes were lysed and probed to examine mTOR expression after IFN-α treatment (**E** and **F**), mTOR deletion after CRISPR (**E** and **G**), and IFITM3 expression by immunoblot (**E** and **H**). β-Actin was used as a loading control. Representative blots are shown. Statistical analysis used was Wilcoxon’s test with values normalized to negative (**G**), Kruskal-Wallis test with values normalized to negative (**F**), and 2-way ANOVA with Šidák’s multiple comparison test with values normalized to negative vehicle (**H**) (*n* = 6–7). *P ≤ 0.05. The β-actin in Figure 2E is the same β-actin as in Supplemental Figure 6A.

We next sought to determine the mechanisms by which IFITM3 expression is regulated in platelets during inflammatory stress. As platelets are anucleate and depend on their parent cell, the megakaryocyte, to regulate gene transcription and, in part, RNA translation and protein synthesis (12), we determined whether IFN-α alters IFITM3 expression in human and murine megakaryocytes. Similarly to platelets, human CD34^+^-derived megakaryocytes express very little IFITM3 protein at baseline. However, upon IFN-α stimulation, IFITM3 protein expression is robustly induced in human megakaryocytes (Figure 2, A–D). In contrast to IFITM3, IFITM1 and IFITM2 proteins are basally expressed in CD34^+^-derived megakaryocytes, but their expression also increases upon IFN-α stimulation (Supplemental Figure 3A). Ifitm3 protein expression in murine megakaryocytes also increases upon IFN-α stimulation (Supplemental Figure 3B). Previous reports have suggested IFN-α activates STAT1 to regulate IFITM3 expression (34). To assess whether STAT1 regulates IFITM3 expression in megakaryocytes, we employed CRISPR/Cas9 technology in human CD34^+^-derived megakaryocytes to delete STAT1 (35). IFN-α stimulation increased STAT1 and phosphorylated STAT1 (p-STAT1) levels in megakaryocytes in a pattern similar to that in IFN-α–induced IFITM3 expression (Figure 2, A–D). STAT1 deletion significantly reduced total STAT1 and p-STAT1 as well as IFITM3 protein expression (Figure 2, A–D). The mTOR and mitogen-activated protein kinase integrating kinase 1 (MNK1) pathways are known to regulate translation of proteins in megakaryocytes and platelets (36, 37). Furthermore, a recent study suggested mTOR regulates IFITM3 expression (38), while another study suggested MNK1 is activated by IFN-α (39). Therefore, we determined whether CRISPR deletion of mTOR or MNK1 altered IFITM3 expression in megakaryocytes. In contrast to STAT1, IFN-α did not alter mTOR or MNK1 expression (Figure 2, E and F, and Supplemental Figure 4). The IFN-α–dependent increase in IFITM3 expression was negated when mTOR was deleted using CRISPR technology, while MNK1 deletion had little effect on IFITM3 expression (Figure 2, E–H, and Supplemental Figure 4). Importantly, STAT1 deletion had no effect on mTOR expression, while mTOR deletion did not change STAT1 levels (Supplemental Figures 5 and 6). Taken together, these data indicate that IFITM3 expression in human megakaryocytes is regulated transcriptionally by STAT1, with translational control occurring through mTOR.

### IFITM3 regulates fibrinogen endocytosis and platelet reactivity.

**Figure 3:**
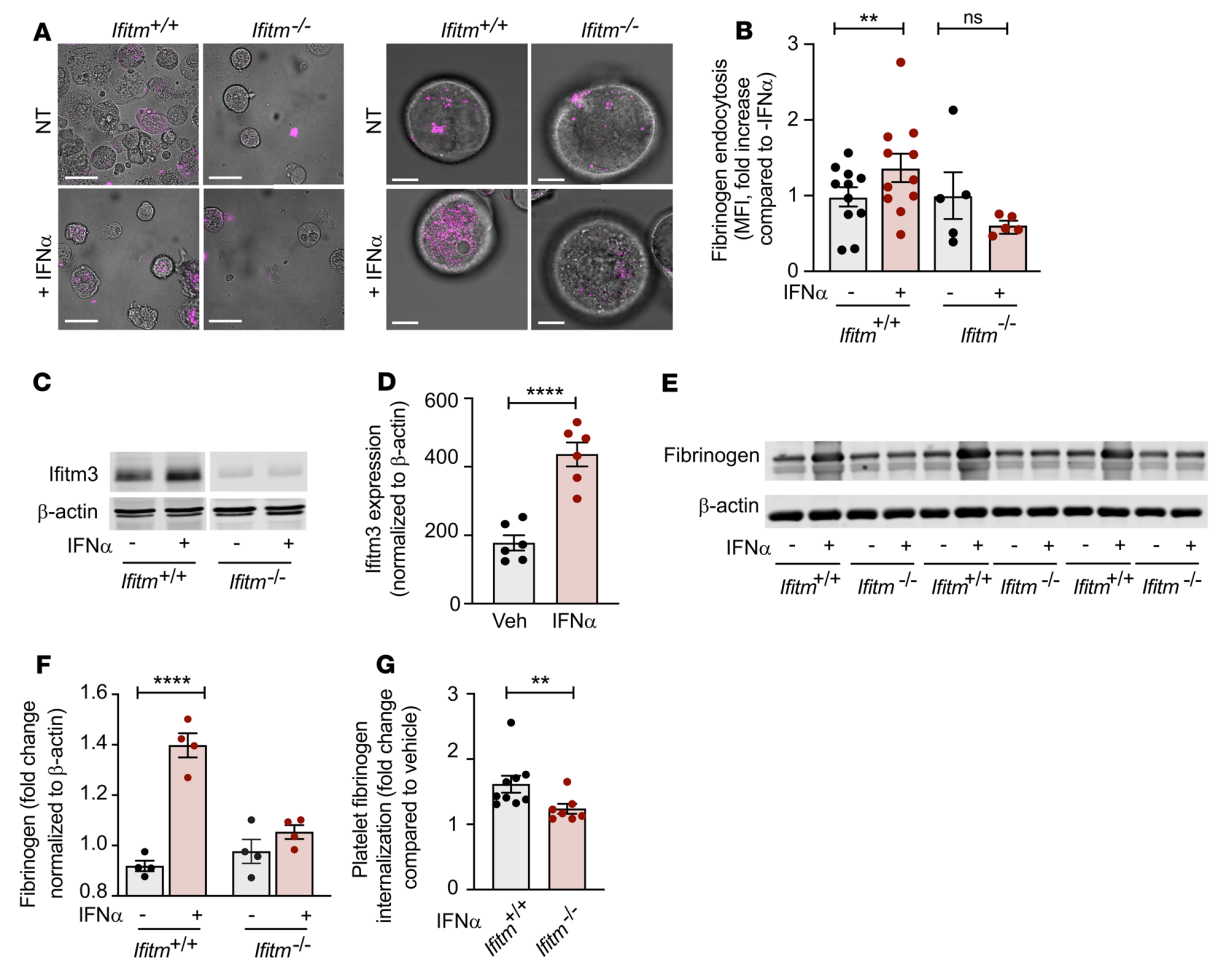
IFITMs regulate fibrinogen endocytosis. (**A** and **B**) Bone marrow megakaryocytes were isolated from *Ifitm^+/+^* and *Ifitm^–/–^* mice and cultured for 4 days before stimulation with IFN-α (1000 U/mL, final) for 24 hours. After 24 hours, 10 μg of Alexa Fluor 546–labeled fibrinogen was added and visualized by immunocytochemistry (**A**). Scale bars: 50 μm (left panels); 10 μm (right panels). The differential contrast interference layer (right side) has been adjusted by eye to create similar contrast for the magenta layer while in Photoshop. The magenta layer brightness contrast was done automatically in FIJI/ImageJ (NIH) before being imported into Photoshop. Images are representative of 3 independent experiments. For quantitative measurements, after 30 minutes of incubation, megakaryocytes were washed with PBS and stained with CD41. CD41- and fibrinogen-positive megakaryocytes were then measured by flow cytometry (**B**). *n* = 5–11. **P ≤ 0.01, mixed-effects analysis with Šidák’s multiple comparisons test. (**C**–**F**) *Ifitm^+/+^* and *Ifitm^–/–^* mice were injected with vehicle (Veh) or murine IFN-α (25,000 U per injection) once daily for 3 consecutive days. Platelets were then isolated and IFITM3 (**C** and **D**) expression and (**E** and **F**) internal fibrinogen stores were measured by Western blot. ****P ≤ 0.0001, unpaired *t* test (**D**) and 1-way ANOVA with Šidák’s multiple comparisons test (**F**). *n* = 6 (**C** and **D**); *n* = 4 (**E** and **F**). (**G**) In vivo fibrinogen endocytosis was examined by injecting *Ifitm^+/+^* and *Ifitm^–/–^* mice with IFN-α as described above, followed by 1 i.v. injection of 100 μg Alexa Fluor 546–labeled fibrinogen. On day 4, whole blood was isolated and the percentage of platelets positive for labeled fibrinogen was measured by flow cytometry. The fold change in positive-labeled fibrinogen platelets is shown compared with that of vehicle-injected *Ifitm^+/+^* and *Ifitm^–/–^* mice (*n* = 7–9 per group). **P ≤ 0.01, Mann-Whitney *U* test.

Previous studies have demonstrated newly synthesized IFITM3 traffics to the plasma membrane, where it participates in endocytosis of viral particles (28). Since platelets and megakaryocytes endocytose immunothrombotic α-granule proteins such as fibrinogen, we next determined whether IFN stimulation altered fibrinogen endocytosis and studied the necessity of IFITM3 for this pathway (18, 40–43). Bone marrow–derived megakaryocytes from *Ifitm^+/+^* and global IFITM-deficient (*Ifitm^–/–^*) mice (27, 44) were cultured and stimulated with IFN-α to increase Ifitm3 expression. In *Ifitm^–/–^* mice, Ifitm1, Ifitm2, and Ifitm3 are all ablated (27, 44). We then monitored endocytosis of fluorescently labeled fibrinogen by immunocytochemistry and flow cytometry. Megakaryocytes from *Ifitm^+/+^* and *Ifitm^–/–^* mice basally endocytosed fibrinogen (Figure 3, A and B). IFN-α stimulation increased fibrinogen uptake only in *Ifitm^+/+^* megakaryocytes, while IFN-α stimulation failed to increase fibrinogen uptake in *Ifitm^–/–^* megakaryocytes (Figure 3, A and B). Similar results were observed with transferrin (Supplemental Figure 7), which also undergoes CME (45). We next asked whether increased IFN-α and IFITM3 altered non-CME and/or phagocytosis of bacterial pathogens and molecules. Similarly to labeled fibrinogen experiments, megakaryocytes from *Ifitm^+/+^* and *Ifitm^–/–^* were incubated with fluorescently labeled *S*. *aureus* (taken up by phagocytosis) or labeled CPG oligodeoxynucleotides (ODNs), short synthetic single-stranded DNA molecules containing unmethylated CPG dinucleotides that undergo clathrin-independent endocytosis (46) in the presence and absence of IFN-α. In contrast to labeled fibrinogen, we observed no difference in the uptake of labeled *S*. *aureus* or CPG ODNs (Supplemental Figure 8). Taken together, our data suggest IFNs and IFITMs selectively regulate the CME of a number of different cargo proteins, including fibrinogen and transferrin, but do not regulate non-CME processes of cargo uptake.

We next examined in vivo fibrinogen endocytosis in circulating platelets sufficient or deficient in IFITMs. To do this, *Ifitm^+/+^* and *Ifitm^–/–^* mice were injected in vivo with IFN-α for 3 consecutive days and platelets were then isolated to measure Ifitm3 expression and endogenous fibrinogen levels. Consistent with our observations in platelets from septic patients, where plasma IFN levels are increased (Figure 1, A–H), and in megakaryocytes stimulated with IFN-α (Figure 2, A–H), the systemic injection of IFN-α increased Ifitm3 expression in platelets from *Ifitm^+/+^* mice (Figure 3C). Similarly to what was seen with Ifitm3 expression, total endogenous fibrinogen levels increased in platelets from IFN-α–stimulated *Ifitm^+/+^* mice, but not in platelets from *Ifitm^–/–^* mice (Figure 3, D–F). To determine whether the uptake of other α-granule cargo in megakaryocyte and/or platelets is altered by IFITMs and/or IFN-α, we examined levels of VEGF, which is synthesized and endocytosed via both clathrin-dependent and clathrin-independent processes by megakaryocytes, and vWF, which is primarily synthesized (47, 48). VEGF and vWF levels were similar between *Ifitm^+/+^* and *Ifitm^–/–^* mice basally and did not significantly change after IFN-α injection (Supplemental Figure 9).

**Figure 4:**
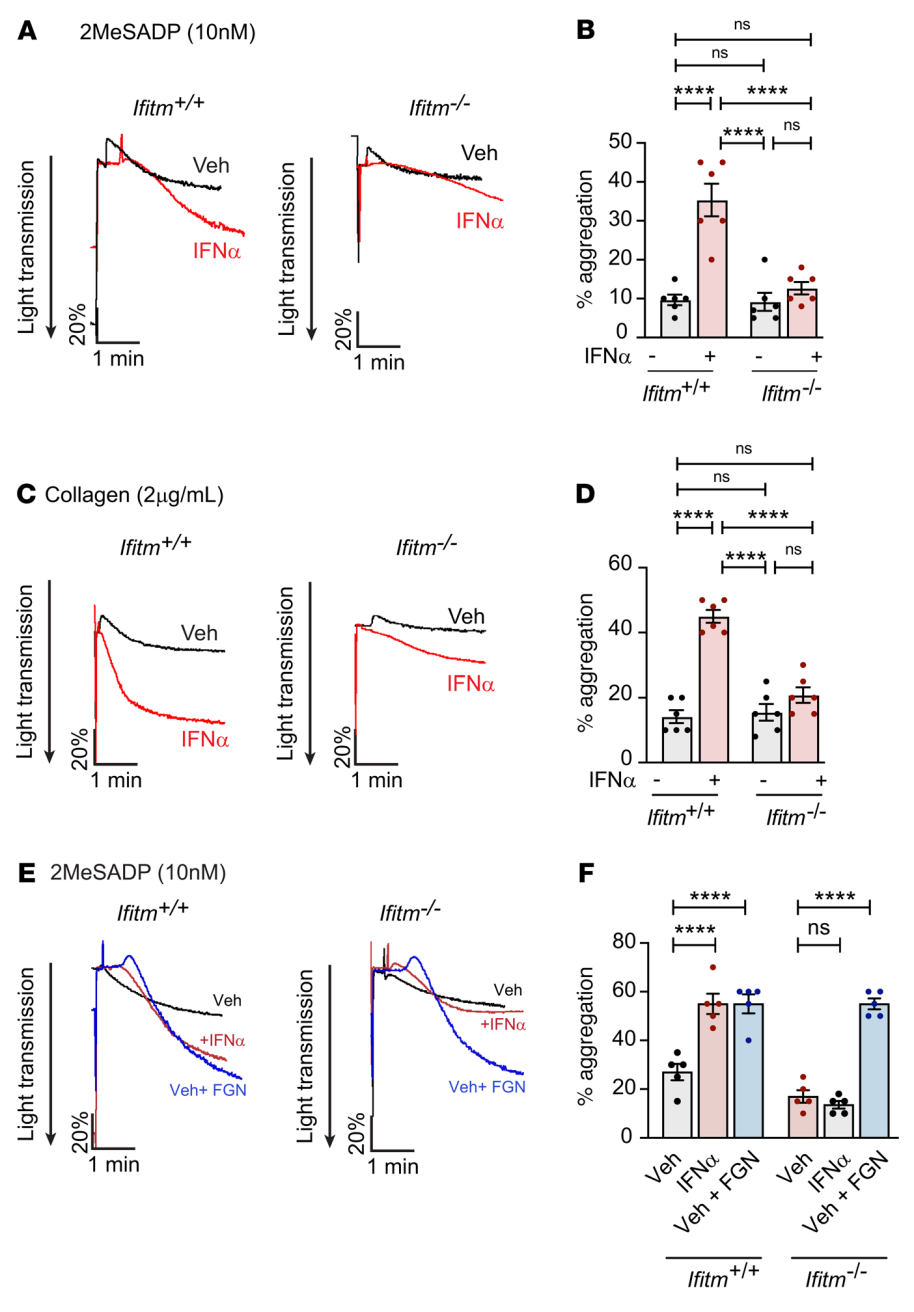
IFITMs regulate platelet hyperactivity. Aggregation was measured in washed platelets from *Ifitm^+/+^* and *Ifitm^–/–^* mice treated with vehicle or murine IFN-α (25,000 U per injection) for 3 consecutive days in response to 2MeSADP (10 nM, final) (**A** and **B**) or collagen (2 μg/mL, final) (**C** and **D**). *n* = 6 per group. ****P ≤ 0.0001, 1-way ANOVA with Tukey’s multiple comparisons test. (**E** and **F**) Aggregation was measured in washed platelets from *Ifitm^+/+^* and *Ifitm^–/–^* mice treated with vehicle or murine IFN-α (25,000 U per injection) for 3 consecutive days in response to 2MeSADP (10 nM, final) with the addition of 10 μg/mL of exogenous fibrinogen (FGN) as indicated. *n* = 5 per group. ****P ≤ 0.0001, 1-way ANOVA with Šidák’s multiple comparisons test.

We next examined in vivo uptake of exogenous, fluorescently labeled fibrinogen. *Ifitm^+/+^* and *Ifitm^–/–^* mice were injected with IFN-α for 3 consecutive days, followed by i.v. administration of fluorescently labeled fibrinogen. Consistent with our previous results, IFN-α injections significantly increased the uptake of labeled fibrinogen by platelets in *Ifitm^+/+^* mice compared with *Ifitm^–/–^* mice (Figure 3G). Of note, IFN-α treatment did not alter α_IIb_ surface expression (Supplemental Figure 10). To address whether increased fibrinogen endocytosis altered platelet function, we performed washed platelet aggregometry to specifically examine the contribution of fibrinogen released from α-granules. IFN-α stimulation in *Ifitm^+/+^* mice significantly increased platelet aggregation in response to adenosine diphosphate (ADP) and collagen stimulation (Figure 4, A–D). However, platelet aggregation was unchanged by IFN-α stimulation in *Ifitm^–/–^* mice (Figure 4, A–D). Increased phosphorylated focal adhesion kinase (p-FAK) activation in platelets from IFN-α–stimulated *Ifitm^+/+^* mice, but not *Ifitm^–/–^* mice, suggested increased fibrinogen binding to α_IIb_β_3_ (Supplemental Figure 11A) (49). The increase in platelet aggregation and p-FAK by IFN-α was independent of changes in total expression of integrin α_IIb_ or β_3_, as IFN-α did not alter protein levels of these 2 integrins (Supplemental Figure 11B). In addition to increased p-FAK in *Ifitm^+/+^* mice treated with IFN, we observed increased p-AKT signaling after collagen-induced platelet aggregation (data not shown). The increase in p-AKT may be linked to IFITM3’s role as a PI3K scaffold (reported previously in B cells), as we observed increased PIP3 levels in the presence of increased IFITM3 expression (50, 51) (Supplemental Figure 12). To further address the role of fibrinogen in potentiating platelet aggregation, we supplemented washed platelet aggregations with increasing concentrations of fibrinogen to determine whether the addition of exogenous fibrinogen could enhance platelet aggregation in *Ifitm^–/–^* mice to levels observed after IFN-α stimulation. Consistent with our previous results (Figure 4, A–D), IFN-α stimulation enhanced platelet aggregation in *Ifitm^+/+^* mice, but not in *Ifitm^–/–^* mice. The addition of 10 μg/mL fibrinogen also enhanced platelet aggregation in *Ifitm^–/–^* mice to levels similar to those observed in IFN-α–stimulated *Ifitm^+/+^* mice (Figure 4, E and F). The addition of fibrinogen also enhanced platelet aggregation in *Ifitm^+/+^* mice in the absence of IFN-α stimulation (Figure 4, E and F, and Supplemental Figure 13). While platelet aggregation was altered after IFN-α treatment in *Ifitm^+/+^* mice, integrin activation (Supplemental Figure 14) was not altered in either vehicle or IFN-α–stimulated *Ifitm^+/+^* and *Ifitm^–/–^* mice. Taken together, these data indicate that IFITMs promote fibrinogen endocytosis and platelet reactivity under IFN-induced inflammatory stress.

### IFITM3 interacts with α_IIb_β_3_ and promotes CME of fibrinogen through alterations in lipid raft domains.

**Figure 5:**
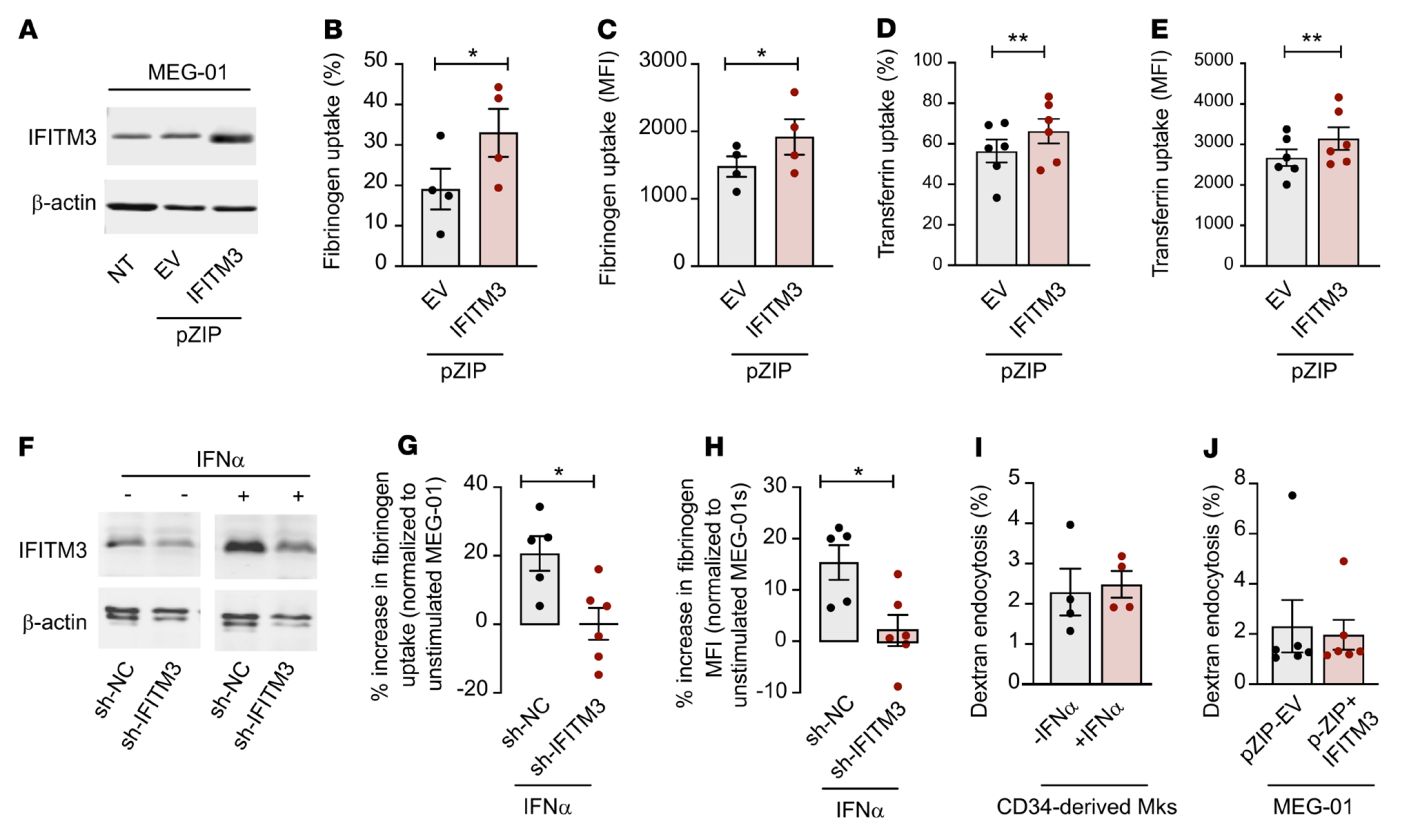
IFITM3 regulates fibrinogen endocytosis in megakaryocytes. MEG-01s were left alone (no pZIP) or transfected with an empty vector control (pZIP+EV) or a vector containing IFITM3 (pZIP-IFITM3). (**A**) IFITM3 expression was determined (representative immunoblot shown from *n* = 3 independent experiments). (**B** and **C**) pZIP EV control or pZIP IFITM3-overexpressing MEG-01s were stimulated with PMA (100 nM, to induce differentiation) for 24 hours before incubation with 10 μg labeled fibrinogen. Fibrinogen endocytosis (percentage and MFI) was measured in mature CD41^+^ MEG-01s by flow cytometry (*n* = 4). Paired *t* test. (**D** and **E**) pZIP EV control or pZIP IFITM3–overexpressing MEG-01s were stimulated with PMA for 24 hours before incubation with labeled transferrin. Transferrin endocytosis (percentage and MFI) was measured in CD41^+^ MEG-01s by flow cytometry (*n* = 6). Paired *t* test. (**F**) MEG-01s were transfected with control shRNA (sh-NC) or a vector containing shRNA against IFITM3 (sh-IFITM3). sh-NC and sh-IFITM3 MEG-01s were treated with IFN-α (1000 U/mL) or vehicle control and IFITM3 measured (representative immunoblot shown from *n* = 4 independent experiments). (**G** and **H**) sh-NC or sh-IFITM3 MEG-01s were stimulated with PMA and IFN-α and incubated with fibrinogen. Fibrinogen endocytosis (percentage change in cells with endocytosed fibrinogen and MFI compared with unstimulated sh-NC or sh-IFITM3 MEG-01) was measured in mature CD41^+^ MEG-01s by flow cytometry (*n* = 5–6). Paired *t* test. (**I**) CD34^+^-derived megakaryocytes (Mks) were treated with vehicle or IFN-α (1000 U/mL) for 24 hours before incubation with labeled dextran. Dextran endocytosis (%) was measured in CD41^+^ megakaryocytes by flow cytometry (*n* = 6). Mann-Whitney *U* test. (**J**) PMA-differentiated MEG-01s were stimulated with IFN-α (1000 U/mL) and 24 hours later incubated with labeled dextran. CD41^+^ MEG-01s were measured for dextran endocytosis by flow cytometry (*n* = 4 per group). Unpaired *t* test. *P ≤ 0.05; **P ≤ 0.01

We next sought to identify the mechanism by which IFITM3 regulates fibrinogen endocytosis. To do this, we first overexpressed IFITM3 in a megakaryocytic cell line, MEG-01s, and examined labeled fibrinogen uptake. MEG-01s express IFITM1, IFITM2, and IFITM3 at various levels endogenously. All 3 IFITMs respond to IFN stimulation, with the highest change observed with IFITM3 (Supplemental Figure 15). Therefore, MEG-01s serve as a relevant cell model for these experiments. Selective overexpression of IFITM3 in MEG-01s significantly increased fibrinogen and transferrin endocytosis in the absence of IFN stimulation (Figure 5, A–E). Fibrinogen endocytosis was dependent on the concentration of exogenous fibrinogen, as we observed a significant increase in endocytosis with 50 μg labeled fibrinogen compared with 10 μg (1,481 ± 151.8 versus 2083 ± 137.7 MFI, *P* = 0.0034) in control MEG-01s. A similar result was observed in IFITM3-overexpressing MEG-01s incubated with 10 μg versus 50 μg labeled fibrinogen (1,919 ± 262.3 versus 2,723 ± 386.9 MFI, *P* = 0.01).

To then establish the necessity of IFITM3 for IFN-dependent increase in fibrinogen uptake, we employed shRNA to specifically knock down IFITM3 only in MEG-01s, while preserving the expression of IFITM1 and IFITM2. In control MEG-01s in which IFITM3 is endogenously present, IFN stimulation increased the expression of IFITM1, IFITM2, and IFITM3 as expected (Figure 5F and Supplemental Figure 15) and significantly upregulated fibrinogen endocytosis (Figure 5, G and H). In contrast, IFN stimulation failed to increase fibrinogen endocytosis in MEG-01s where IFITM3 was knocked down, indicating IFITM3 was necessary to regulate endocytosis in the presence of IFN-α (Figure 5, G and H). These data also suggest that IFITM1 and IFITM2 are unable to rescue fibrinogen endocytosis in the absence of IFITM3. This increase in fibrinogen endocytosis was mediated by fibrinogen binding to α_IIb_β_3_ rather than fibrinogen binding to IFITM3, as RGDS (which inhibits binding of fibrinogen to integrin α_IIb_β_3_; ref. 52) blocked the uptake of fibrinogen (Supplemental Figure 16).

To address whether other forms of cellular uptake, such as pinocytosis, were regulated by IFITM3, we used pharmacologic (e.g., IFN-α) and genetic (e.g., overexpressing) tools to increase IFITM3 expression in CD34^+^-derived megakaryocytes and MEG-01s. Cells were incubated with fluorescent dextran (which undergoes pinocytosis), and dextran uptake was measured. Dextran pinocytosis was unaltered in IFN-α–stimulated CD34^+^-derived megakaryocytes or IFITM3-overexpressing MEG-01s (Figure 5, I and J), indicating that IFITM3 regulates CME but not pinocytosis.

**Figure 6:**
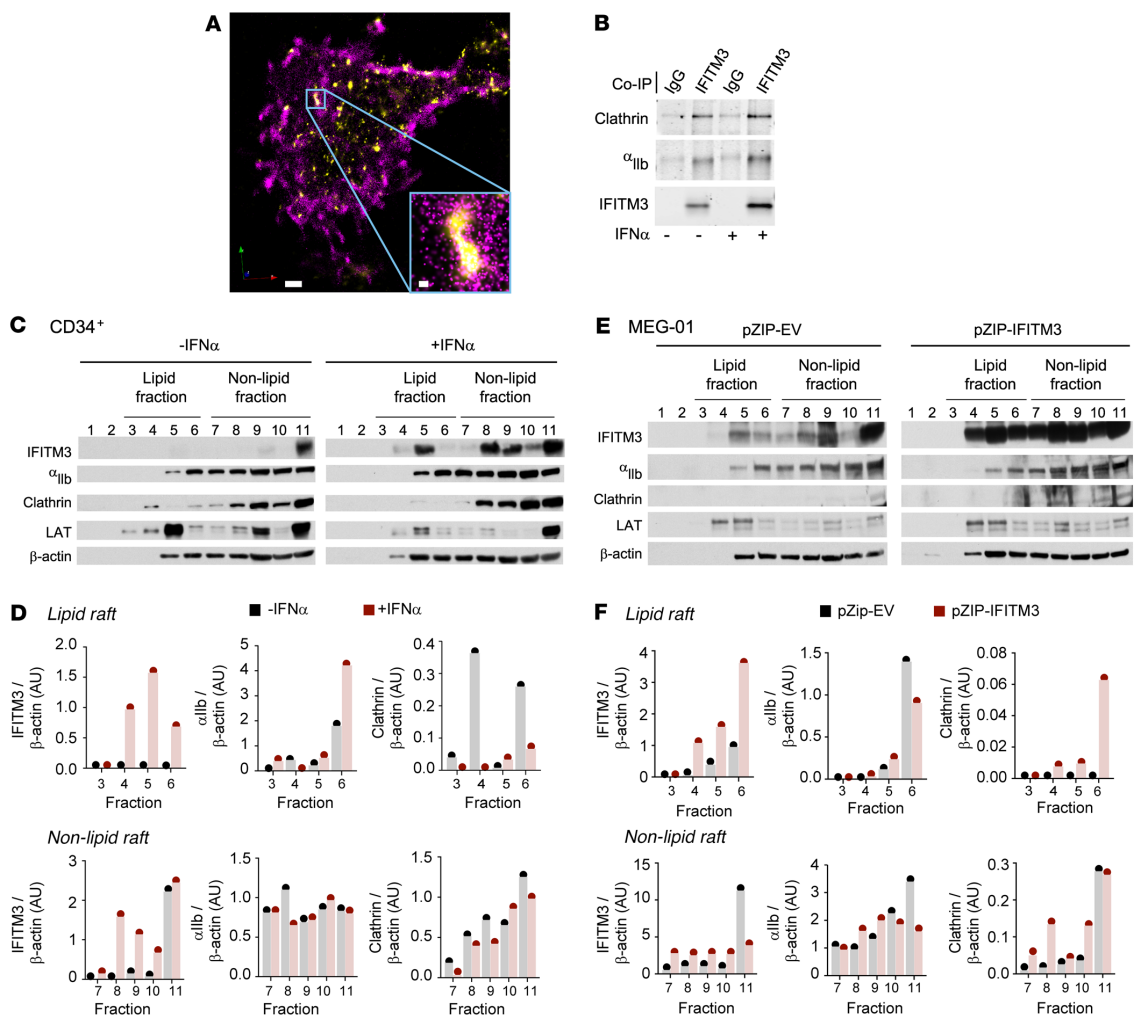
IFITM3 alters α_IIb_ and clathrin localization into IFITM3-expressing lipid rafts. (**A**) Bone marrow–derived, murine megakaryocytes were immunostained with anti-IFITM3 (magenta) and anti-α_IIb_ (yellow) antibodies. Cells were subsequently analyzed using super-resolution microscopy. Scale bars: 1 μm. Figure is representative of *n* = 3 independent experiments. (**B**) CD34^+^-derived megakaryocytes were stimulated with vehicle or IFN-α (1000 U/mL, final). IFITM3 was immunoprecipitated and α_IIb_ and clathrin binding to IFITM3 were examined by immunoblot in comparison with IgG control. Representative immunoblot from 4 independent experiments. (**C** and **D**) CD34^+^-derived megakaryocytes were stimulated with vehicle or IFN-α (1000 U/mL, final) and fractionated using a sucrose density gradient to isolate lipid rafts. Representative immunoblots for IFITM3, α_IIb_, clathrin, LAT (a lipid raft specific marker), and β-actin, a loading control, are shown in **C**, and the representative blots are quantified in **D**. Exposures were the same for each protein pair. Fraction 11 contains the insoluble cellular material from the lysis and density gradients (*n* = 3 independent experiments). (**E** and **F**) MEG-01s were transfected with an empty vector control (pZIP+EV) or an engineered vector containing IFITM3 (pZIP-IFITM3). MEG-01s were lysed and fractionated using a sucrose density gradient to isolate lipid rafts. Representative immunoblots for IFITM3, α_IIb_, clathrin, LAT, and β-actin are shown in **E** and quantified in **F**. Fraction 11 contains the insoluble cellular material from the lysis and density gradients (*n* = 3 independent experiments). Exposures were the same for each protein pair.

As fibrinogen endocytosis is classically mediated through integrin α_IIb_β_3_ (41–43), we next performed superresolution microscopy on murine, proplatelet-producing megakaryocytes and observed that α_IIb_ and IFITM3 colocalize with each other (Figure 6A). Consistent with this, IFITM3 coimmunoprecipitation studies in CD34-derived, human megakaryocytes identified α_IIb_ and clathrin as IFITM3-binding partners (Figure 6B). Interestingly, clathrin and α_IIb_ binding to IFITM3 appeared to increase in the presence of IFN-α (Figure 6B). A recent publication suggested IFITM3 is necessary for lipid raft formation in B cells (50). In platelets, lipid rafts are enriched with CD36, Src, and Lyn and are critical membrane microdomains for platelet activation and prothrombotic cellular responses (53, 54). In addition, α_IIb_β_3_ and clathrin both localize in lipid rafts to mediate fibrinogen endocytosis (30). However, whether IFITM3 regulates the localization of α_IIb_β_3_ and clathrin to lipid rafts in megakaryocytes and platelets is unknown. Lipid rafts were isolated from IFN-α–treated or untreated control CD34^+^-derived human megakaryocytes. In the absence of IFN-α stimulation, α_IIb_ and clathrin were expressed at low levels in isolated lipid rafts containing IFITM3, as indicated by expression of linker of activated T cells (LAT), a marker of lipid rafts (55) (Figure 6, C and D). IFN-α stimulation shifted IFITM3 into lipid rafts and α_IIb_ and clathrin associated with IFITM3 containing lipid rafts. Importantly, IFN-α treatment did not alter expression of α_IIb_, clathrin, or LAT (Supplemental Figure 17). As IFN-α may directly alter localization of α_IIb_ and clathrin into lipid rafts independently of IFITM3, we then studied MEG-01s in which IFITM3 was genetically and selectively overexpressed. Overexpression of IFITM3 in MEG-01s, independent of IFN stimulation, similarly shifted α_IIb_ and clathrin into IFITM3-containing lipid rafts (Figure 6, E and F). Overexpressing IFITM3 did not alter expression of α_IIb_, clathrin, or LAT (Supplemental Figure 18). These results highlight a role for IFITM3 in megakaryocyte and platelet biology by regulating localization of α_IIb_ and clathrin into lipid rafts, areas of cellular activation often triggering procoagulant responses.

### IFITM-mediated platelet hyperactivity contributes to thrombosis.

**Figure 7:**
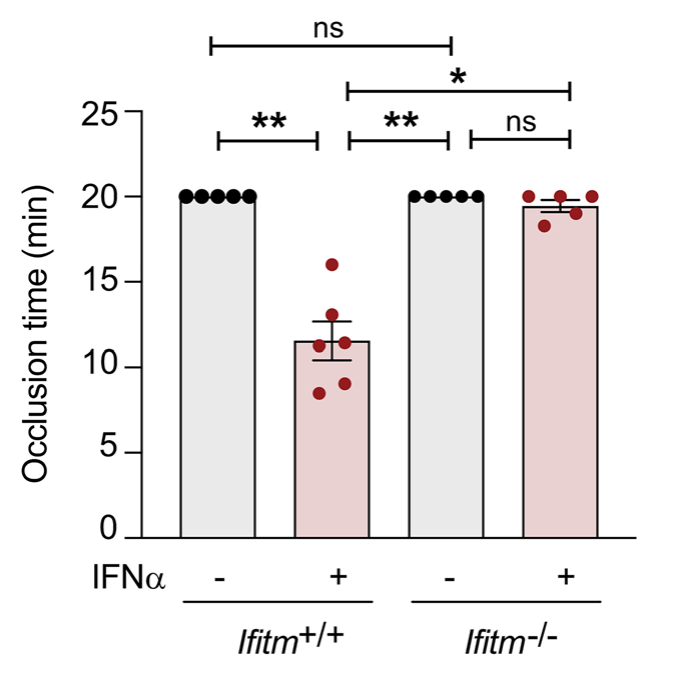
IFITMs regulate platelet-dependent thrombosis in vivo. *Ifitm^+/+^* and *Ifitm^–/–^* mice were injected with vehicle or murine IFN-α (25,000 U per injection) once daily for 3 consecutive days. On day 4, mice were anesthetized and the right carotid artery was exposed. Thrombosis was induced by topical application of a filter paper soaked in 10% FeCl_3_ for 3 minutes. Excessive FeCl_3_ was rinsed with PBS and blood flow was measured downstream of the application site with a laser Doppler flow probe and monitored for 20 minutes. *n* = 5–6 per group. *P ≤ 0.05; **P ≤ 0.01, Kruskal-Wallis test with multiple comparisons test.

As IFITM3 enhances fibrinogen endocytosis and aggregation (Figure 3, Figure 4, and Figure 5), we next asked whether IFN-α increased thrombosis in an IFITM-dependent mechanism in vivo. *Ifitm^+/+^* and *Ifitm^–/–^* mice were injected with IFN-α for 3 consecutive days to increase IFITM3 expression. After 3 days, mice were subjected to 2 different thrombosis models: a ferric chloride carotid injury model and a collagen-epinephrine thrombosis model, which are both dependent on platelets occluding blood flow either in the carotid artery (56, 57) or the microvasculature in the lungs (58). *Ifitm^+/+^* mice stimulated with IFN-α had significantly faster occlusion times in the ferric chloride thrombosis model (Figure 7). However, vehicle- and IFN-α–stimulated *Ifitm^–/–^* mice had occlusion times similar to those of vehicle-treated *Ifitm^+/+^* mice (Figure 7). In the collagen-epinephrine pulmonary thrombosis model, *Ifitm^+/+^* mice had significantly faster times to death compared with vehicle-treated *Ifitm^+/+^* mice (Supplemental Figure 19). Vehicle- and IFN-α–stimulated *Ifitm^–/–^* mice had similar mortality rates, indicating IFITMs mediate in vivo thrombosis during settings of IFN-α–induced inflammatory stress (Supplemental Figure 19).

### Upregulation of platelet IFITM3 during clinical sepsis is associated with increased fibrinogen endocytosis and enhanced platelet reactivity.

**Figure 8:**
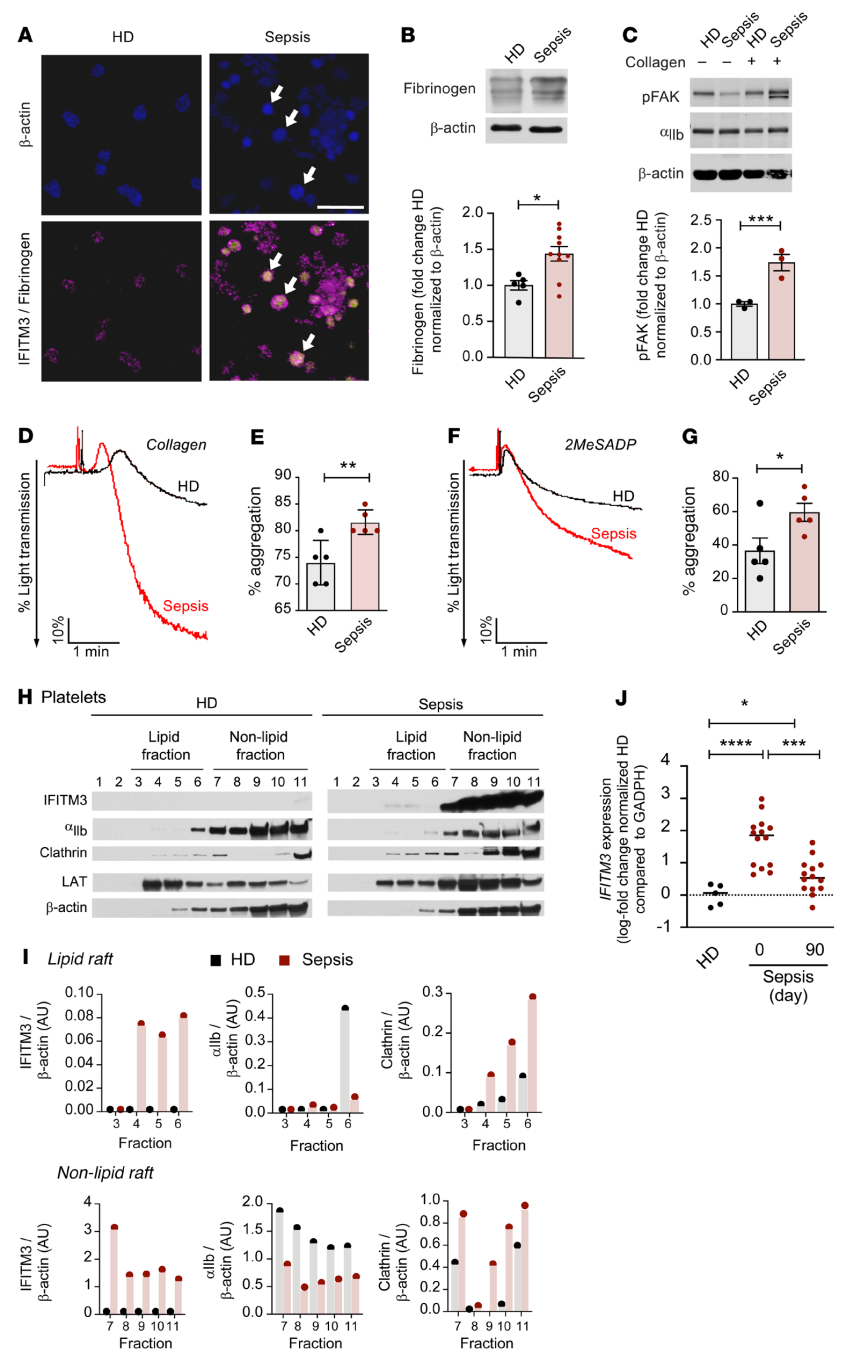
Increased IFITM3 expression in nonviral sepsis patients is associated with increased fibrinogen content and platelet hyperreactivity. (**A**) Platelets from healthy donors and nonviral sepsis patients were stained for IFITM3 (yellow), fibrinogen (magenta), and actin (blue). White arrows demonstrate representative platelets positive for IFITM3 and fibrinogen (*n* = 3). Pink arrows indicate IFITM3-positive, fibrinogen-positive platelets. Scale bars: 1 μm. (**B** and **C**). Immunoblot of platelets isolated from healthy donors and nonviral sepsis patients and probed for fibrinogen, α_IIb_, and p-FAK in the presence or absence of activation by collagen (2 μg/mL, final) (*n* = 3). Unpaired *t* test. (**D**–**G**) Washed platelet aggregation using platelets isolated from healthy donors and nonviral sepsis patients in response to collagen (2 μg/mL, final) and 2MesADP (10 nM, final) (*n* = 5 per group). Unpaired *t* test. (**H** and **I**) Platelets isolated from healthy donors and nonviral sepsis patients were fractionated for lipid rafts using a sucrose density gradient and probed for IFITM3, α_IIb_, clathrin, LAT, and β-actin. Representative blots are shown in **H** and quantified in **I**. Exposures for each protein pair were the same. Fraction 11 contains all the nonsoluble material (*n* = 3 independent experiments). (**J**) Platelet mRNA was isolated from healthy donors (*n* = 5) and nonviral sepsis patients (*n* = 14) at day 0 (initial hospitalization) and from the same sepsis patients at day 90 (days after discharge). *IFITM3* expression was measured by quantitative reverse-transcriptase PCR (RT-qPCR) normalized to *GAPDH* and compared with expression levels in healthy donors. Brown-Forsythe and Welch’s ANOVA tests with Dunnett’s T3 multiple comparisons test. *P ≤ 0.05; **P ≤ 0.01; ***P ≤ 0.001; ****P ≤ 0.0001.

Our clinical observations indicate that patients with nonviral sepsis have increased platelet IFITM3 expression (Figure 1). However, whether the increase in IFITM3 is associated with increased fibrinogen endocytosis and platelet reactivity in septic patients is unknown. Platelets from nonviral septic patients and healthy donors were isolated and phenotyped. Similarly to platelets from IFN-α–stimulated control mice, platelets from septic patients (where IFITM3 is increased) had significantly greater fibrinogen content than those from healthy donors (Figure 8, A and B). Consistent with fibrinogen being an acute phase reactant, sepsis patients had increased plasma fibrinogen levels (Supplemental Figure 20). Moreover, expression of fibrinogen and IFITM3 proteins in platelets from septic patients often colocalized (Figure 8A). Collagen- and ADP-induced washed platelet aggregations were significantly increased in septic patients compared with healthy donors (Figure 8, D–G), consistent with our findings in IFN-α–challenged mice. Increased platelet aggregation in septic patients was independent of changes in α_IIb_ total expression (Figure 8C and Supplemental Figure 21) but similarly to our findings in mice, was associated with increased phosphorylation of FAK, a kinase activated downstream of β_3_ (Figure 8C).

We next determined whether increased platelet IFITM3 expression in septic patients was associated with α_IIb_ and clathrin associating with IFITM3-containing platelet lipid rafts. Platelet lipid rafts isolated from healthy donors had some expression of α_IIb_ and clathrin and no readily detectable IFITM3. In comparison, IFITM3-expressing platelet lipid rafts from septic patients were associated with α_IIb_ and clathrin (Figure 8, H and I). Importantly, α_IIb_, clathrin and LAT expression were similar between healthy donors and sepsis patients (Supplemental Figure 21). As patients with sepsis are at increased risk for longer-term thrombotic complications even after their sepsis has resolved (10, 11), we longitudinally examined platelet IFITM3 expression acutely in septic patients and again, 90 days later, in the same septic patients who had recovered. Interestingly, while lower than during acute sepsis, the expression of IFITM3 RNA remained significantly elevated in recovered septic patients compared with healthy donors, suggesting sepsis may trigger durable changes to the platelet transcriptome (Figure 8J).

### Sepsis-induced platelet hyperreactivity in mice is regulated by IFITM3.

**Figure 9:**
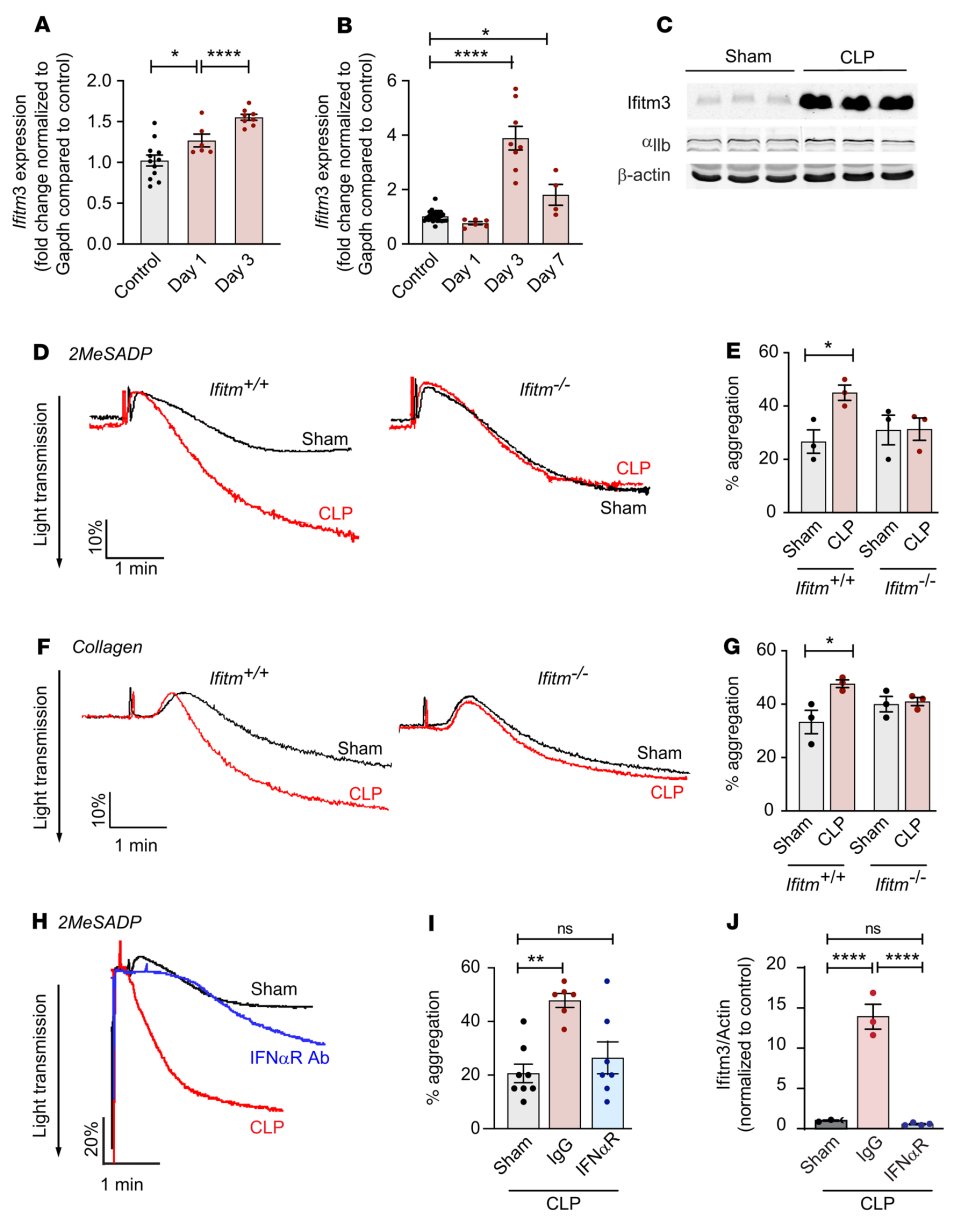
IFITM deficiency reduces platelet reactivity in murine model of sepsis. (**A** and **B**) Sepsis was induced in *Ifitm^+/+^* mice by CLP, and *Ifitm3* expression in flow-sorted CD41^+^ bone marrow megakaryocytes and CD45-depleted platelets was examined at the indicated times by RT-PCR. *Ifitm3* expression was normalized to *Gapdh* and compared with that control mice. *n* = 6–12 (**A**); *n* = 5–27 (**B**). *P ≤ 0.05; ****P ≤ 0.0001, 1-way ANOVA with Dunnett’s post hoc test compared with control. (**C**) Platelet Ifitm3 protein expression in sham or CLP *Ifitm^+/+^* mice was examined by immunoblot at day 3 of CLP. *n* = 3. (**D**–**G**) Sepsis was induced in *Ifitm^+/+^* and *Ifitm^–/–^* mice by CLP, and platelets were isolated at day 3 after CLP for wash platelet aggregation in response to 2MesADP (10 nM, final **D** and **E**) and collagen (2 μg/mL, final **F** and **G**). Platelet aggregation was compared with that of sham-operated control in either *Ifitm* *^+/+^* or *Ifitm* *^–/–^* mice (*n* = 3 per group). *P ≤ 0.05, 1-way ANOVA with Šidák’s multiple comparisons test. (**H**–**J**) Sepsis was induced in *Ifitm^+/+^* mice by CLP. One hour before and 6 hours after CLP, mice were treated with 1 mg (total, i.p.) of either an anti–IFN-αR1 antibody or control IgG. Washed platelets were isolated at day 3 after CLP and aggregation assessed in response to 2MesADP. (**H** and **I**). Platelet Ifitm3 expression was also measured by immunoblot (Supplemental Figure 23). (**J**). One-way Kruskal Wallis test with Dunn’s multiple comparisons test or 1-way ANOVA with Tukey’s multiple comparisons test (**J**). **P ≤ 0.01; ****P ≤ 0.0001. *n* = 6–8 (**H** and **I**); *n* = 3–4 (**J**).

Given our clinical findings in septic patients (Figure 1 and Figure 8), we assessed whether experimental bacterial sepsis induces platelet Ifitm3 expression and increases platelet reactivity using a murine polymicrobial cecal-ligation and puncture (CLP) model (20). CLP-induced sepsis increased *Ifitm3* mRNA expression in primary murine bone marrow megakaryocytes within 24 hours after CLP, and expression remained elevated in megakaryocytes through day 3 (Figure 9, A and B). In circulating murine platelets, *Ifitm3* mRNA expression did not increase until day 3 following CLP, but remained elevated through day 7 (Figure 9, A and B). Consistent with the increase in *Ifitm3* mRNA at day 3 after CLP, a significant increase in Ifitm3 protein was also observed in platelets following CLP sepsis (Figure 9C). This suggest that sepsis-induced increases in platelet IFITM3 is driven, at least in part, through signaling delivered to, and sensed by, bone marrow megakaryocytes. Interestingly, CLP increased the expression of integrin α_IIb_ protein (Figure 9C), a finding we previously observed (20).

We next asked whether or not CLP induced changes in platelet reactivity and, if so, whether these changes were mediated by IFITMs. *Ifitm^+/+^* and *Ifitm^–/–^* mice underwent sham or CLP surgery, and platelet aggregation was measured in washed platelets. CLP induced a significant increase in systemic IFN-α levels in both *Ifitm^+/+^* and *Ifitm^–/–^* mice, while little change in IFN-γ was observed (Supplemental Figure 22), consistent with our findings in septic patients (Figure 1F and Supplemental Figure 1). Consistent with our clinical data in septic patients, CLP significantly enhanced collagen- and ADP-induced aggregation in *Ifitm^+/+^* mice compared with sham-operated animals. In contrast, in the absence of *Ifitms*, CLP did not enhance platelet aggregation (Figure 9, D–G). To assess the specific role of IFN-α in mediating this response, we injected *Ifitm^+/+^* mice with control IgG or anti–IFN-αR IgG, an antibody against type 1 IFN receptors, 1 hour before and 6 hours after CLP (59). Consistent with what is shown in Figure 9, D–G, CLP increased platelet reactivity in *Ifitm^+/+^* mice compared with sham-operated animals (Figure 9H). Treating *Ifitm^+/+^* mice with anti–IFN-αR IgG after CLP blunted platelet aggregation responses (Figure 9, H and I) and platelet IFITM3 expression (Figure 9J and Supplemental Figure 23). This suggests that during experimental sepsis, increased platelet Ifitm3 expression and aggregation is mediated by IFN-α.

## Discussion

Emerging evidence indicates megakaryocytes and platelets are critical players in immune responses during viral and bacterial infections (13–20). For example, megakaryocytes express and present antigens through MHC-I–dependent mechanisms to activate T cells (15). Additionally, megakaryocytes and platelets express a wide variety of antimicrobial and antiviral peptides that are released upon activation, many of which regulate immune and inflammatory responses (14). While megakaryocytes and platelets are equipped to fight infections, inflammation can alter the repertoire of genes and proteins expressed, resulting in altered cellular functions. Previously, our group demonstrated that viral infections such as dengue and influenza increase systemic IFNs, which then upregulate the expression and function of IFITM3 in megakaryocytes and platelets (19). Importantly, increased IFITM3 expression limits viral replication in megakaryocytes and prevents infection in surrounding stem cells, indicating that these transcriptional and translational changes in megakaryocytes can be beneficial to the host. Nevertheless, whether IFITM3 regulates the endocytosis of nonviral hemostatic cargo, thereby orchestrating platelet functional responses, remains completely unknown.

The present study found that the platelet transcriptome and proteome are dynamically altered during nonviral sepsis, consistent with previous reports by our group in viral sepsis (19, 21). This includes the upregulation of classical antiviral genes such as IFITM3, even in the absence of viral infections. The increase in IFITM3 was primarily driven by increased IFNs, which were capable of activating the STAT1 transcriptional and mTOR translational pathways to induce synthesis of IFITM3. Our studies focused on IFITM3 because this gene was one of the top transcriptionally and translationally upregulated genes in septic patients. While IFITM3 mRNA and protein were upregulated in sepsis patients, IFITM3 protein expression negatively correlated with systemic IFN-α levels, suggesting IFN levels increase early in sepsis, followed by transcriptional and translational changes in the megakaryocyte consistent with the kinetics of Ifitm3 expression changes in our murine studies. Given the increases in both IFITM3 mRNA and protein during nonviral sepsis, we speculated that IFITM3 may have noncanonical roles in hemostasis and thrombosis during nonviral sepsis, a concept not examined previously, to the best of our knowledge, in megakaryocytes or platelets. While the mechanism of action remains incompletely understood, IFITM3 resides in late endosomes and lysosomes, blocking viral cytosolic entry through either altering the rate of viral fusion with endosomes and/or trafficking viral particles to lysosomes for destruction (60). In addition to regulating endocytic cargo fusion with endosomes and lysosomes, IFITM3 also has been shown to regulate rates of receptor turnover, such as endothelial growth factor receptor (EGFR) (60). As IFITM3 is known to regulate these biological processes, we asked whether IFITM3 could regulate the entry of hemostatic proteins important to megakaryocyte and platelet functional responses.

As platelets are anucleate, they depend on the megakaryocyte to synthesize and invest a majority of the proteins found in them. However, megakaryocytes are not responsible for synthesizing all proteins found in platelets. Proteins such as fibrinogen (41–43), transferrin (45), and immunoglobulins (61, 62) are all endocytosed by platelets instead of being synthesized. As IFITM3 can regulate endocytosis and thrombosis is a complication of bacterial sepsis, we asked whether increased IFITM3 also increased the endocytosis of procoagulant platelet proteins during septic conditions. Inflammation induced by IFN increased IFITM3 expression on mouse and human megakaryocytes and resulted in increased fibrinogen and transferrin endocytosis. Selective, genetic deletion of IFITM3 in human and mouse megakaryocytes blunted the inflammation-induced increase in endocytosis. Furthermore, IFITM3 overexpression in the absence of IFN increased endocytosis, demonstrating the necessity and sufficiency for IFITM3 in endocytosis. To further determine whether IFITM3 alters additional α-granule cargo, we investigated whether IFN-α and IFITM3 altered VEGF or vWF levels. Previous studies have suggested VEGF can be synthesized and endocytosed while vWF is synthesized in megakaryocytes (47, 48). VEGF uptake can be clathrin independent (63) or dependent (64, 65) depending on the cell type, and VEGF uptake remains incompletely understood in platelets (66). Interestingly, neither VEGF nor vWF levels were altered by IFN-α nor by the presence or absence of IFITM. In addition, as platelets and megakaryocytes play critical immune roles, we asked whether uptake of bacterial pathogens was altered by IFITM3. Phagocytosis of *S*. *aureus* or uptake of CPG ODN, which is mediated by clathrin-independent endocytosis (46, 67), was not altered by IFITM3 expression. Finally, we examined other forms of cellular uptake and observed pinocytosis was not affected by the presence or absence of IFITM3. Taken together, these data indicate that IFITM specifically regulates cargo uptake by platelets and megakaryocytes via CME.

We observed similar increases in platelet fibrinogen content in vivo after injecting mice with IFNs to increase IFITM3 expression. As IFNs could alter circulating fibrinogen content, which could directly impact fibrinogen uptake, we also examined labeled fibrinogen endocytosis. Consistent with our findings with endogenous fibrinogen uptake, labeled fibrinogen endocytosis was increased when IFITM3 expression was increased after in vivo IFN administration. Importantly, during inflammatory stress, both endogenous fibrinogen and labeled fibrinogen endocytosis were almost completely blocked when IFITM3 was absent, identifying IFITM3 as a regulator of endocytosis in platelets and megakaryocytes. As endocytosis is conserved across many cell types in addition to platelets (68), we speculate that this function of IFITM3 may have broad relevance beyond platelets and megakaryocytes.

Functionally, the IFITM3-dependent increase in fibrinogen endocytosis enhanced platelet aggregation and subsequent downstream activation of β_3_ integrins. In mice, IFN administration increased platelet aggregation in response to ADP and collagen, while mice lacking IFITM3 had little change in aggregation responses after IFN treatment. To demonstrate the importance of exogenous fibrinogen release in mediating the response, the addition of fibrinogen to *Ifitm^–/–^* platelets rescued IFITM-dependent platelet aggregation. Several previous studies have indicated platelet aggregation may be reduced during sepsis, which may seem inconsistent with our present findings (69–71). However, one distinct difference in our study is that all platelet aggregation assays were performed with washed platelets to remove any potential confounding effects from plasma components. This also allowed us to more specifically examine fibrinogen released by platelet α-granules, excluding the effects of any circulating plasma fibrinogen, and to mechanistically demonstrate that this cellular compartment of fibrinogen is critical during inflammation and sepsis. Furthermore, this cellular pool of fibrinogen increased downstream β_3_ signaling, as observed by p-FAK, indicating increased fibrinogen uptake and release enhances platelet signaling (49). Our data also suggest the potential for IFITM3 to serve as a PI3K scaffold, resulting in enhanced AKT signaling. This may serve as an additional regulator of platelet activation (50, 51), but additional studies are needed to fully elucidate and separate the role of IFITM3 in endocytosis from AKT signaling.

To determine whether there was a relevant in vivo function for the upregulation of platelet IFITM3, we utilized 2 platelet-dependent thrombosis models (56, 58, 72). Under inflammatory stress, increased IFITM3 expression was associated with greater arterial thrombosis and mortality from pulmonary thromboembolism. Similarly to what occurred with ex vivo platelet aggregation, IFN did not increase thrombosis in the absence of IFITM3, additional evidence that IFITM3 is a critical regulator of platelet activation and thrombosis during inflammatory stress. Sepsis patients are also at increased risk for thrombotic complications even after sepsis has resolved. Our findings that platelet IFITM3 mRNA remains elevated 90 days after discharge in recovered sepsis patients implicate IFITM3 as a potential regulator of postsepsis thrombosis. These data indicate ongoing systemic inflammation or changes to the bone marrow milieu after sepsis may continue to alter platelet transcriptome and function.

Mechanistically, IFITM3 possesses an YxxΦ-sorting motif that enables IFITM3 to regulate endocytosis through binding to the μ2 subunit of the AP-2 complex, which binds clathrin (27, 28). Furthermore, IFITM3 and clathrin colocalize in lipid rafts, which is also critical in regulating endocytosis (27, 50). While previous studies have demonstrated global deletion of IFITMs alters lipid raft and clathrin localization, a direct mechanistical link for IFITM3 regulating these critical cellular processes has not been demonstrated. To determine whether IFITM3 altered these pathways in megakaryocytes and platelets, we performed superresolution microscopy and coimmunoprecipitation and observed that the fibrinogen receptor α_IIb_, clathrin, and IFITM3 closely interact with each other. Using pharmacological and genetic approaches, we observed that when IFITM3 was upregulated, IFITM3 associated with α_IIb_ and clathrin in platelet lipid rafts containing IFITM3, which could help facilitate increased fibrinogen uptake. Our data indicate the increase in fibrinogen uptake is independent of changes in the level of α_IIb_ or clathrin expression. We also observed this shift of α_IIb_ and clathrin in platelet lipid rafts expressing IFITM3 from septic patients. In comparison, in platelets from healthy donors, IFITM3 was low to absent from lipid rafts. This finding demonstrates that inflammation can dramatically alter the localization of platelet proteins into lipid raft microdomains. These findings may have implications in sepsis and other diseases in which IFITM3 expression is increased (e.g., cancer; ref. 73).

Our platelet transcriptomic and proteomic data indicate other IFITMs, including IFITM1 and IFITM2, are also increased in sepsis and IFN-induced inflammation. This is expected, as both IFITM1 and IFITM2 are IFN responsive. We also appreciate that IFITM1 and IFITM2 are deleted in our mouse model. Furthermore, the current work is limited due to the lack of a cell-specific IFITM3 knockout. Nevertheless, we do not believe these are limitations to our findings. Our experimental evidence suggests that IFITM3 specifically regulates fibrinogen endocytosis, as IFITM3 is the only IFITM that has an YxxΦ-sorting motif that is required for CME. Moreover, specific IFITM3 genetic silencing and overexpression studies (in which IFITM1 and IFITM2 were unaffected) demonstrated IFITM3 is necessary and sufficient for fibrinogen endocytosis. Finally, the use of ex vivo platelet assays and platelet-dependent thrombosis models further supports a role for IFITM-dependent platelet vascular thrombosis.

These findings may have implications for other disease states in which IFN levels are increased and vascular thrombosis is common, such as malignancy and severe, systemic viral infections (e.g., COVID-19 and influenza A/H1N1) (19, 21, 74). Interestingly, platelets from patients with multiple myeloma or myeloproliferative disorders had significantly increased IFITM3 expression (Supplemental Figure 24), suggesting IFITM3 may also promote thrombotic complications in these patients: a hypothesis to be tested in future studies. Previously, we observed increased IFITM3 in influenza and COVID-19 patients (19, 21). It is also interesting to speculate as to whether during severe viral infections, the upregulation of IFITM3 in platelets and megakaryocytes may serve dual roles (e.g., immune and hemostatic functions) as other proteins, such as fibrinogen, in platelets are known to do (18, 40). Further studies are warranted to better understand the role of IFITM3 in these inflammatory settings.

In conclusion, nonviral sepsis upregulates IFITM3 in platelets and megakaryocytes. Our observations indicate that IFITM3 interacts with α_IIb_ and clathrin and alters their localization to lipid rafts. This shift in localization increases CME of fibrinogen and other immune-thrombotic proteins endocytosed by megakaryocytes and platelets. The resulting enrichment of cellular and surface-bound fibrinogen promotes platelet reactivity and microvascular thrombosis. These findings identify a role for IFITM3 in regulating megakaryocyte and platelet functions and further support the concept that inflammation-induced changes to the platelet transcriptome and proteome can alter platelet behavior, host responses, and outcomes.

## Methods

### Study participants.

Critically ill patients with sepsis (*n* = 45) were recruited from the Medical Intensive Care Unit at the University of Utah Health Sciences Center in Salt Lake City between September 2016 and October 2019. Sepsis was defined using the consensus criteria at the time this study was actively recruiting, systemic infection and 2 or more of the following: (a) temperature greater than 38°C or less than 36°C; (b) heart rate greater than 90 beats/min; (c) respiratory rate greater than 20 breaths/min or PaCO_2_ less than 32 mm Hg; and (d) white blood cell count greater than 12,000 × 10^9^/L, less than 4,000 × 10^9^/L, or greater than 10% bands. The prescription of antiplatelet agents (aspirin at any dose, clopidogrel, or nonsteroidal antiinflammatory drugs) was recorded based on medication reconciliations done by medical staff upon ICU admission. All patients underwent clinically directed investigations to identify the pathogen causing infection, including bacterial, respiratory, and urinary cultures, and antigen testing, as directed by the treating clinical team. As is common in sepsis patients, only approximately 35% of patients had a pathogen specifically identified. Pathogens that were identified included *E*. *coli* (*n* = 6/45), *Streptococcus* (*n* = 5/45), *Staphylococcus* (*n* = 3/45), yeast (*n* = 2/45), and *Klebsiella pneumoniae* (*n* = 1/45) (Supplemental Table 4). In addition to recording the pathogen, when identified, we also tracked the clinically identified site or organ of the primary infection. These included urosepsis (*n* = 14/45, 31%), pneumonia (*n* = 11/45, 24%), skin and soft tissue infection (*n* = 11/45, 24%), other (*n* = 3/45, 7%), or unknown (*n* = 6/45, 13%) (Supplemental Table 5). All patients were enrolled within 72 hours of ICU admission. Healthy age- and sex-matched donors (*n* = 23) were also enrolled. Healthy donors had no known bleeding disorder, liver or kidney disease, cancer, or history of surgery or thrombotic event within the past 3 months and were not on antiplatelet or anticoagulant therapy. Clinical laboratory values (platelet counts, white blood cell counts) were assessed by ARUP Laboratories (Salt Lake City,Utah, USA). Reference ranges as of August 2019 were provided by ARUP Laboratories. To examine the platelet transcriptome acutely and during recovery in a subset of septic patients, a previous cohort of septic samples was used and platelet *IFITM3* expression was measured from septic patients (*n* = 14) upon study enrollment (e.g., acutely, after ICU admission for sepsis) and again in the same subjects approximately 90 days after enrollment (e.g., recovery from sepsis) (20). In these longitudinal studies, age- and sex-matched, independently recruited, healthy donors (*n* = 5) were enrolled for comparison. RNA-Seq data were deposited in the NCBI’s Gene Expression Omnibus database (GEO GSE210797) and can be found in Supplemental Table 3.

### Ifitm^+/+^ and Ifitm^–/–^ mice.

Mice lacking the *Ifitm* genes (*Ifitm* *1*, *2*, *3*, *5* and 6 from chromosome 11) (*Ifitm^–/–^*) were obtained from John H. Weiss (University of Utah) and were previously backcrossed to C57BL/6J mice (27, 44). *Ifitm^–/–^* were further backcrossed to C57BL/6J mice (Jackson Laboratory) to generate *Ifitm^+/+^* and *Ifitm^–/–^* mice, which were used for all subsequent experiments. Animals used in these experiments were male and female mice age 8 to 14 weeks.

### Murine in vivo IFN injections.

Male and female mice of 8 to 12 weeks were injected (i.p.) with 25,000 units total murine IFN-α or vehicle control (100 μL, total) for 3 consecutive days. On day 4, mice were anesthetized with ketamine and xylazine and blood harvested by cardiac puncture. Platelets were isolated as described above. In some experiments, fibrinogen from human plasma (Alexa Fluor 488 conjugate,100μg, total) was injected by tail vein on day 3. On day 4, whole blood was isolated by cardiac puncture, diluted 1:50 into Tyrode’s buffer, and platelets stained with CD41-APC. Platelet-specific labeled fibrinogen endocytosis was then measured by flow cytometry. Samples were immediately run on a BD CytoFLEX.

### Murine model of sepsis: CLP.

Male and female mice at 8 to 12 weeks of age were anesthetized with isoflurane. An incision was made at the left lower quadrant, the cecum was identified, and ligation was performed distal to the ileocecal junction. A single puncture was carefully made in the cecum with an 18-gauge needle to allow expression of fecal content. The intestine was returned within the peritoneum, and the incision was closed with a 3-0 silk. Immediately postoperatively, mice were given 0.5 ml sterile saline s.c. for fluid resuscitation. There was no survival difference between *Ifitm^+/+^* and *Ifitm^–/–^* mice (data not shown). On day 3, platelets were isolated from *Ifitm^+/+^* and *Ifitm^–/–^* mice by cardiac puncture, and platelet aggregation was performed as described above. In some experiments, *Ifitm^+/+^* mice were treated, 1 hour before CLP and 6 hours after CLP, with 1 mg (total, i.p.) of an anti–IFN-aR1 antibody or control IgG. Platelets were isolated at day 3 for platelet aggregation studies and to determine platelet IFITM3 expression as measured by immunoblot.

Further information can be found in Supplemental Methods.

### Statistics.

Continuous variables from all experiments were assessed for normality with skewness and kurtosis tests. Data that were normally distributed were expressed as mean ± SEM. For analyses involving 2 groups, parametric 2-tailed Student’s *t* test was used. When 3 or more groups were analyzed, ANOVA with Tukey’s post hoc test was performed. When data were not normally distributed, Mann-Whitney *U* test was used when 2 groups were analyzed, while Kruskal-Wallis with Dunn’s multiple comparison post hoc test was used for analyses of 3 or more groups. When appropriate, 2-way ANOVA with post hoc test was used as described. Summary statistics were used to describe the study cohort, and clinical variables were expressed as the mean ± SEM or as a number and percentage (%). Statistical analyses were performed using GraphPad Prism (version 9). A 2-tailed *P* value of less than 0.05 was considered statistically significant.

### Study approval.

Each study participant or their legally authorized representative gave written, informed consent for study enrollment in accordance with the Declaration of Helsinki. All patients were recruited under protocols approved by the IRB of the University of Utah (IRB 00102638). Healthy age- and sex-matched donors were enrolled under a separate protocol (IRB 0051506). Animal experiments detailed in this study were approved by the University of Utah IACUC (protocols 18-10012 and 21-09012).

## Author contributions

BKM designed and performed a significant number of experiments, and MB helped with a significant portion of the revision. RAC, BKM, MB, EAM, AA, HS, FD, EM, NDT, SS, LP, CKG, SMB, DWS, AK, and MTR designed and performed experiments. RAC, BKM, MB, DL, CS, and MTR analyzed results and made the figures. RAC and MTR wrote the manuscript. All authors reviewed and critically edited the manuscript. First author order was determined as follows: RAC conceived and designed the experiments in collaboration with MTR; RAC also wrote the manuscript; BKM performed experiments for initial submission and MB assisted with experiments for revision.

## Supplementary Material

Supplemental data

Supplemental table 2

Supplemental table 3

## Version 1 10/04/2022

In-Press Preview

## Version 2 12/01/2022

Electronic publication
