# Supplementary material for: IFITM3 regulates fibrinogen endocytosis and platelet reactivity in nonviral sepsis
Source: J Clin Invest. 2022 Dec 1;132(23):e153014. doi: 10.1172/JCI153014 (PMC9711880; doi:10.1172/JCI153014)
Supplement: Supplemental data [file jci-132-153014-s010.pdf]

# IFITM3 Regulates Fibrinogen Endocytosis and Platelet Reactivity in Non-Viral Sepsis


Robert A. Campbell[1,2,3*#], Bhanu Kanth Manne[1*], Meenakshi Banerjee[1*], Elizabeth A. Middleton[1,2], Abigail Ajanel[3], Hansjorg Schwertz[1,4,5], Frederik Denorme[1], Chris Stubben[6], Emilie Montenont[1], Samantha Saperstein[1], Lauren Page[1], Neal D. Tolley[1], Diana Lim[1], Samuel M Brown[7], Colin K. Grissom[7], Douglas W. Sborov[8], Anandi Krishnan[9,10], Matthew T. Rondina[1,2,3,11#]

[1]University of Utah Molecular Medicine Program, Salt Lake City, Utah, 84112
[2]Department of Internal Medicine, University of Utah, Salt Lake City, Utah, 84132
[3]Department of Pathology, University of Utah, Salt Lake City, Utah, 84132
[4]Rocky Mountain Center for Occupational and Environmental Health, University of Utah, Salt Lake City, Utah, 84132
[5]Occupational Medicine, Billings Clinic Bozeman, Bozeman, MT 59715
[6]Bioinformatics Shared Resource, Huntsman Cancer Institute, University of Utah, Salt Lake City, UT, USA, 84132
[7]Division of Pulmonary and Critical Medicine, Department of Medicine, Intermountain Medical Center, Murray, Utah, 84107.
[8]Huntsman Cancer Institute, University of Utah, Salt Lake City, UT, USA, 84132
[9]Stanford Cancer Institute, Stanford University School of Medicine, Stanford, CA, 94305
[10]Department of Pathology, Stanford University, Stanford, CA, 94305
[11]George E. Wahlen VAMC Department of Internal Medicine and GRECC, Salt Lake City, Utah, 84148
[*]Indicates shared contribution
#Indicates co-corresponding authors


Running Title: IFITM3 Regulates Platelet Reactivity


COI: Matthew Rondina has a patent on platelet transcriptomics.



Co-Corresponding Author:

Robert Campbell, PhD
Assistant Professor of Medicine and Pathology
University of Utah
Eccles Institute of Human Genetics
15 North 2030 East, Bldg 533 RM 4280
Salt Lake City, Utah, 84112
rcampbell@u2m2.utah.edu

Matthew T. Rondina, MD, MS
Professor of Medicine and Pathology
University of Utah Health Science Center
Eccles Institute of Human Genetics
15 North 2030 East, Bldg 533 RM 4280
Salt Lake City, Utah 84112
Matthew.rondina@hsc.utah.edu


Abstract: 197
Word Count: 11952
Number of Figures: 9
Number of Supplemental Figures: 24
Number of Supplemental Tables: 5

**Supplemental Methods**

*Reagents and Antibodies*

2MeSADP was from Tocris. Apyrase (type VII) was from Sigma. Collagen was from Chrono-log. Epinephrine was from Helena. Fibrinogen From Human Plasma, Alexa Fluor™ 488 and 647 Conjugate, Transferrin from Human Serum, Alexa Fluor™ 488 Conjugate (T13342), Transferrin from Human Serum, Alexa Fluor™ 568 Conjugate (T23365), and Dextran, Alexa Fluor™ 555; 10,000 MW (D34679), Staphylococcus aureus (Wood strain without protein A) BioParticle and Alexa Fluor™ 488 conjugate (S23371) were from ThermoFisher. FITC ODN 2335 (CPGs, tlrl-2395f) were from Invivogen. Whatman protein nitrocellulose transfer membrane was from Fisher Scientific. LI-COR Odyssey blocking buffer was from LI-COR Biosciences. Rabbit anti-IFITM1 (Proteintech, 60074-1 Ig), rabbit anti-IFITM2 (Proteintech, 12769-1 AP), and rabbit anti-IFITM3 (Proteintech, 11714-I AP) antibodies used for immunoprecipitation and western blots was from Proteintech. A mouse anti-human clathrin antibody (BD Biosciences 610499), a rabbit anti-LAT antibody (Cell Signaling 9166S), a rabbit anti-$\beta3$ integrin antibody (Proteintech, 18309-1-AP), a rabbit anti-$\alpha$IIb antibody (Cell Signaling, 13807), a rabbit anti-Phospho-FAK (Tyr397) antibody (Cell Signaling, 3283S), a rabbit anti-fibrinogen antibody (Dako, A0080), a rabbit anti-Phospho-Stat1 (Tyr701) antibody (Cell Signaling, 9167), rabbit anti-total STAT1 antibody (Cell Signaling, 9172), a rabbit anti-MNK1 antibody (Cell Signaling, 2195), a rabbit ant-mTOR antibody (Cell Signaling, 2983), and a mouse anti-actin antibody (Abcam, ab20272) were used for western blots and immunoprecipitation. Rabbit anti-IFITM3 antibody (Abcam, ab74699), mouse anti-fibrinogen antibody (Abcam, ab119948), a rat anti-CD41a Antibody (ThermoFisher, MWReg30), and anti-GPIb$\beta$ derivative (Emfret, X488) were used for immunocytochemistry and super resolution microscopy. Cyclic-RGDS (AS-61111) was from AnaSpec. Rapamycin (553210) was from Calbiochem. Murine IFN$\alpha$ was from Miltenyi Biotec (130-093-130). Human IFN$\alpha$ (IF007) was from Sigma Aldrich. For flow cytometry, murine platelets were labeled with CD41 APC (ThermoFisher, MWReg30). For activation assays, platelets were labeled with JON/A PE from Emfret (M023-2) and were activated with human thrombin (Sigma-Aldrich, T-1063) or convulxin (Santa Cruz, SC-202554). For human megakaryocytes and MEG-01 flow cytometry experiments, cells were labeled with CD41 (559777) from BD Biosciences. Anti-IFN$\alpha$R (BE0241) and IgG control (BE0083) antibodies were from Bioxcell.

*Human Platelet isolation*

Whole blood was collected from septic patients and healthy donors into acid- citrate-dextrose (ACD, 3.2%) sterile tubes. Leukocyte-depleted platelets were isolated as previously described(1-6). Briefly, the whole blood was first centrifuged at 150xg for 20min at room temperature (RT). The platelet-rich plasma (PRP) was removed and PGE1 was added to the PRP to prevent exogenous platelet activation. The PRP was then centrifuged at 400xg for 20min at RT. The platelet pellet was re-suspended in warmed (37°C) PIPES saline glucose (PSG). Leukocytes were depleted using CD45+ magnetic beads (Miltenyi Biotec). In select studies,

glycophorin A beads were used to remove erythrocytes (Miltenyi Biotec). Isolated platelets were either re-suspended in Tyrodes Buffer or lysed in buffer (TRIZOL, or Laemmli) for designated experiments.

*Murine platelet isolation*

Whole blood was drawn from 8–12-week mice by cardiac puncture into 10% ACD. Platelet isolation was performed as previously described(2, 3, 7, 8). In brief, whole blood was diluted to 2mL with warmed (37°C) PSG and centrifuged at 150xg for 10min to separate the PRP. The PRP was removed and PGE1 was added to the PRP to prevent exogenous platelet activation. The PRP was then centrifuged at 400xg for 10min at RT. A second wash with warmed PSG in the presence of PGE1 was performed. For select experiments, leukocytes and were depleted using CD45+ (Miltenyi Biotec). Platelets were then either re-suspended in Tyrodes Buffer or lysed in buffer (TRIZOL or Laemmli) for designated experiments.

*RNA Preparation and Sequencing*

Illumina TruSeq Stranded mRNA libraries were prepared, and 50 base pair paired end reads sequenced on a NovaSeq 6000 sequencer. The human GRCh38 genome and gene feature files were downloaded from Ensembl release 98 and a reference database was created using STAR version 2.7.2c with splice junctions optimized for 50 base pair reads. Optical duplicates were removed from the paired reads using clumpify v38.34 and adapters were trimmed using cutadapt 1.16. The trimmed reads were aligned to the reference database using STAR in two pass mode to output an aligned BAM file sorted by coordinates. Mapped reads were assigned to annotated genes in the gene feature file using featureCounts version 1.6.3.

RNA-seq and analysis were performed on total RNA extracted from platelets isolated from non-viral sepsis patients and age-, and gender-matched healthy donors, as before(3-5, 9). The normalized readcount ratio of PTPRC/ITGA2B (pan leukocyte marker CD45 gene PTPRC/platelet marker gene ITGA2B), which was used as an index of leukocyte mRNA contamination, was less than 0.001 and equivalent between control and sepsis samples (p=0.71). Raw counts were filtered before running DESeq2 to remove features with zero counts, five or fewer reads in every sample and all non-coding genes.

*Quantitative Real-Time PCR (qRT-PCR)*

qRT-PCR was utilized to validate RNA-Seq findings in a cohort of prospectively recruited septic patients (n=4) and age and gender matched healthy donors (n=7). An additional cohort of septic patients (n=16) and had qRT-PCR performed at day 1 and day 90 following enrollment along with age and sex-matched healthy donors (n=5). In select experiments, qRT-PCR was performed on murine platelets and isolated bone marrow megakaryocytes as described below. Either iScript (Bio-Rad) or Superscript II (ThermoFisher) was utilized for IFITM3 qRT-PCR. Relative mean fold changes and propagated standard errors were calculated according to the 2(−ΔΔCt) method. B2M and GAPDH were chosen as housekeeping transcripts as their expression was not altered in sepsis. Forward and reverse human primers were as follows, respectively: (1) IFITM3: 5’-CTAGGGACAGGAAGATGGTTGG-3’and 5’- ATCCATAGGCCTGGAAGATCAG-3, (2) *B2M*: 5’-AGATGAGTATGCCTGCCGTGT-3’ and 5’-AGCTACCTGTGGAGCAACCTG-3, and (3) GADPH 5’-

GAACATCATCCCTGCCTCTACTG-3’ and 5’-AGCTTGACAAAGTGGTCGTTGAG-3’. Forward and reverse murine primers were as follows, respectively: (1) *Ifitm3*: 5’- CCCCCAAACTACGAAAGAATCA-3’ and 5’-ACCATCTTCCGATCCCTAGAC-3’ and (2) *Gadph*: 5’-AGGTCGGTGTGAACGGATTTG-3’ and 5’-GGGGTCGTTGATGGCAACA-3’.

*STAT1, MNK1 and mTOR CRISPR/Cas9 transfection*

CD34$^+$ cells were transfected with CRISPR/Cas9 complexes on day 2 (mTOR) or day 5 (STAT1 and MNK1) of culture using minor modifications of published protocols to accommodate our MK culture conditions(10). Predesigned Alt-R CRISPR/Cas9 CRISPR RNAs (crRNA) with >65% on-target score were purchased from Integrated DNA Technologies (IDT, Coralville, IA). IDT negative control #1 was used as a nontargeting control. All materials and reagents were kept sterile and RNAse free throughout. crRNA, trans-activating CRISPR RNA (tracrRNA) (IDT #1072532), and electroporation enhancer (IDT #1075915) were resuspended to 100 μM in 10 mM tris(hydroxymethyl)aminomethane (Tris; pH 7.5), and stored at −20°C until use. crRNA and tracRNA were mixed 1:1 (2.5 μL each) in a polymerase chain reaction (PCR) tube and heated at 95°C for 5 minutes in a thermal cycler with a heated lid, followed by cooling to room temperature on a bench top for at least 10 minutes before storing at −20°C. Immediately before transfection, RNP complexes were formed by mixing (gentle swirling with pipet tip) the following in a PCR tube: for 2 cords, 2.1 μL of phosphate-buffered saline (PBS; no calcium or magnesium), 1.2 μL of crRNA-tracRNA duplex, and 1.7 μL Alt-R *Streptococcus pyogenes* (S.p.) (Alt-R S.p.) Cas9 V3 (62 μM stock, IDT #1081058). The mixture was heated in a thermal cycler at 37°C for 4 minutes and allowed to cool to room temperature on a bench top for 10 minutes. Simultaneously, cells were counted, washed once with PBS (centrifuged at 200*g* at room temperature), and suspended at 0.25 × $10^6$ to 4 × $10^6$ cells per transfection in a PCR tube containing 20 μL of room temperature Amaxa nucleofector solution P3 (Lonza, Basel, Switzerland). Then, 2.5 μL of RNP complex (for double CRISPR co-transfections, use 2.5 μL of each) was gently mixed with 20 μL of cells and 1 μL of enhancer and transferred to a 16-well electroporation strip. Cells were transfected using Amaxa nucleofector 4D program DZ100. Immediately following transfection, 75 μL of prewarmed media (SFEM with TPO-stem cell factor) was added to the cuvette, left to sit for 5 minutes at room temperature, then gently transferred to a 24-well plate containing 500 μL of media. Finally, 24 hours later, cells were passaged and culture was continued as described above. On day 13, CD34$^+$-derived megakaryocytes were treated in with or without of IFN$\alpha$ (500 or 1000 U/mL, final) for 24 hours and then harvested to probe for expression of STAT1, pSTAT1, mTOR, MNK1, and IFITM3.

*Super-resolution microscopy.*

Super-resolution microscopy was performed as previously described(11). Using this technique, two-point sources closer than the width of their point source function (PSF) can be distinguished. Having only a few labeling molecules in a fluorescent state (blinking function) at any time allows the location of each molecule to be individually determined with high precision. Therefore, this technique is primarily aimed at defining a location instead of intensity of fluorophore. Platelets were pretreated with rapamycin or bafilomycin A as aforementioned. Cells were fixed in suspension using paraformaldehyde (PFA, 2% final concentration). The

fixed cells were spun down onto poly-L-lysin coated chamber slides using standard centrifugation, and custom slide holder. Cells were washed (HBSS, 3x), and permeabilized using HBSS/Triton-X 0.1% for 5 minutes. Cells were washed (HBSS, 3x), and non-specific binding blocked using HBSS containing filtered donkey serum (10%, abcam, ab7475) for 1 hr. at room temperature. Cells were subsequently incubated with primary antibodies (rabbit anti-IFITM3 antibody, and rat anti-$\alpha$IIb) at 4°C overnight. Next, samples were incubated with secondary antibodies: goat anti-rabbit Alexa 647, and donkey anti-rat Cy3b (1:1,000, 1 hr. at room temperature, in-house custom labelling). After additional washing steps, cells were post-fixed using 4% paraformaldehyde for 10 minutes at room temperature. Samples were imaged using a Bruker Vutara SR200 Biplane 3D microscope (Bruker, Middleton, WI) equipped for single molecule localization microscopy (SMLM). Images were analyzed using the proprietary software, Vutara SRX software, and results transferred to Adobe Photoshop CS6, and ImageJ (NIH).

*Megakaryocyte and platelet immunocytochemistry.*

Freshly isolated platelets were fixed in suspension using PFA, permeabilized with 0.1% Triton-X for 5 min. at room temperature (RT) and subsequently layered onto VECTABOND™ (Vector Laboratories, SP1800) coated coverslips using a cytospin centrifuge (Shandon Cytospin, Thermo Fisher Scientific, Waltham, MA). Cells were incubated with IgG (to control for specificity of signal) or an antibody against targets as listed above (see reagents and antibodies paragraph) over night at 4°C. Cells were subsequently incubated with secondary biotinylated antibodies, and signals were amplified using streptavidin Alexa Fluor® 488 or 546 respectively. Sialic acids/glycoproteins (in cell membranes/granular content) and the actin cytoskeleton were co-stained using WGA or phalloidin, respectively. Murine megakaryocytes were fixed with 4% PFA and allowed to settle by gravity onto glass chamber slides. Labeled-fibrinogen uptake was imaged by confocal microscopy. Fluorescent fibrinogen uptake was determined by comparing vehicle (absence of labeled fibrinogen) treated megakaryocytes to megakaryocytes incubated with labeled fibrinogen.

*Microscopy and image analysis.*

High-resolution confocal reflection microscopy was performed using an Olympus IX81, FV300 (Olympus, Melville, NY) confocal-scanning microscope equipped with a 60x/1.42 NA oil objective for viewing platelets and megakaryocytes. An Olympus FVS-PSU/IX2-UCB camera and scanning unit and Olympus Fluoview FV 300 image acquisition software version 5.0 were used for recording. Monochrome 16-bit images were further analyzed and changes quantified using Adobe Photoshop CS6, and ImageJ (NIH).

IFN ELISAs

Whole blood was collected from each subject using a 21-g needle vacutainer butterfly into acid/citrate/dextrose-anticoagulant. Whole blood was centrifuged (150x*g*, 20 minutes) to generate platelet-rich plasma. Platelet-rich plasma was then centrifuged (1,500x*g*, 20 minutes) to produce platelet-poor plasma, which was flash frozen in liquid nitrogen and stored at -80°C. Plasma from patients with sepsis and healthy donors were isolated in a similar manner (blood draws, centrifugations, and time from blood draw to freezer).

Plasma IFN levels were then measured by ELISA (Human IFN$\alpha$ (BMS6027) and Human IFN (EHIFNG), Thermofisher). For murine studies, 24 hours after CLP, blood was collected by cardiac puncture and plasma isolated by centrifuging blood for 10 minutes at 2000 x g. Plasma was isolated and frozen by liquid nitrogen and stored at -80°C. Plasma IFN levels were then measured by ELISA (Murine Mouse IFN$\gamma$ (BMS606) and Mouse IFN$\alpha$ (BMS6027), Thermofisher).

*Western blotting*

Megakaryocytes and platelets were treated as described and then treated with 0.6 N $HClO_4$ to precipitate the protein. The resulting acid precipitate was collected and kept on ice. The samples were centrifuged at 13 000 × *g* for 4 minutes followed by re-suspending in 0.5 mL of deionized water. The protein was again pelleted by centrifugation at 13 000 × *g* for 4 minutes. The protein pellets were solubilized in sample buffer containing 0.1 mol/L Tris, 2% sodium dodecyl sulfate (SDS), 1% (v/v) glycerol, 0.1% bromophenol blue, and 100 mmol/L dithiothreitol (DTT), and then boiled for 10 minutes. Proteins were resolved by SDS polyacrylamide gel electrophoresis and transferred to nitrocellulose membranes (Whatman Protran). Membranes were blocked with Odyssey blocking buffer for 1 hour at ambient temperature, incubated overnight at 4°C with the desired primary antibody, and then washed four times with tris-buffered saline Tween 20 (TBS-T). Membranes were incubated with the appropriate secondary infrared dye-labeled antibody for 60 minutes at room temperature and washed four times with 10% TBS-T. Membranes were examined with a Li-Cor Odyssey infrared imaging system.

*MEG-01 transfection and endocytosis*

MEG-01s (ATCC, CRL-2021) were generated in a previous study(9). The pZIP-mEF1a vector (shERWOOD-UltramiR Lentiviral shRNA) containing green fluorescent protein (GFP) was purchased from Transomics (Huntsville, Alabama). IFITM3 was cloned into the pZIP empty vector by replacing GFP using single blunt end ligation which destroyed the AgeI but maintained the EcoRI sites. Plasmids were purified by Qiagen MaxiPrep kits (Germantown, Maryland) and sequenced over the modified region to ensure proper placement and orientation of IFITM3. Verified plasmids were packaged into lentivirus by System Biosciences (Palo Alto, California). MEG-01s (ATCC, Manassas, Virginia) cells were transduced using an MOI of 50 plus polybrene for both IFITM3 and empty constructs. Medium was exchanged 24 hours after transduction. To select positively transduced cells, puromycin was added to growth medium for one week. Cells were then allowed to grow without puromycin for an additional week. Twenty-four hours before endocytosis experiments, MEG-01s were differentiated with the addition of 100 nM PMA. After 24 hours, Alex-Fluor 647 labeled fibrinogen (10 or 50 $\mu$g, total) added to the MEG-01 for 30 minutes and then the cells were washed with three times PBS. Mature megakaryocytes were labeled with CD41 and fibrinogen endocytosis was measured by flow cytometry. In some experiments, Alexa-Fluor 546 transferrin (50 $\mu$g, total) was incubated for 30 minutes before the cells were washed and imaged by flow cytometry. MEG-01s were also treated with dextran Alexa Fluor 555; 10,000 MW (0.5 mg/mL, final) for 30 minutes to measure pinocytosis. After 30 minutes, cells were three times washed and

dextran pinocytosis measured by flow cytometry. All samples were measured on a BD Cytoflex. For PIP3 (Echelon Biosciences, K-2500S) and PIP2 (Echelon Biosciences, K-4500) ELISAs, >45 million cells per a condition were used. Samples were isolated and split in half to measure PIP3 and PIP2 levels as described previously(12).

*VEGF and VWF ELISA*

*Ifitm*$^{+/+}$ and *Ifitm*$^{-/-}$ mice were injected with vehicle or murine IFNα (25,000 U per injection) once daily for three consecutive days. Platelets were then isolated and total VEGF levels (EMVEGFACL, ThermoFisher) and vWF levels (Abcam, ab208980) were measured by ELISA.

*IFITM3 Immunoprecipitation*

CD34$^+$-derived megakaryocytes were treated with vehicle or 1000 U/mL IFNα on day 12. On day 13, cells were centrifuged at 500 x g for 10 minutes and washed with PBS. Cells (5 x10^6, total) per condition were then lysed using a Pierce Crosslink Magnetic Co-Immunoprecipitation Kit (88805). To co-immunoprecipitate IFITM3, IFITM3 (Proteintech, 11714-I AP) or Rabbit IgG (ThermoFisher) were conjugated to magnetic protein A/G beads and crosslinked to the beads for 60 minutes at room temperature. After 60 minutes, the beads were washed and added to the total cell lysate (5 x10^6, total cells) at 4$^o$C for 24 hours. After 24 hours, the beads were washed and boiled for western blot analysis. Samples were run on SDS-Page gels and probed for IFITM3, clathrin, and αIIb.

*Bone Marrow Derived Megakaryocytes*

Mouse bone marrow cells were collected from 8–12-week-old mice and cultured at 37°C and 5% $CO_2$ in the presence of supernatant containing 70 ng/mL recombinant mouse c-Mpl ligand for 4 days. On day 3, megakaryocyte cultures were treated vehicle or 1000 U/mL (final) murine IFNα. On day 4, round MKs were isolated by BSA gradient sedimentation. In some experiments, megakaryocytes were lysed and probed for IFITM3. In other experiments, purified megakaryocytes were treated with 10 or 50 μg total Alexa-Fluor labeled fibrinogen or vehicle for 30 minutes. Megakaryocytes were then washed three times with PBS and then labeled with MWReg30 (ThermoFischer) to identify CD41$^+$ megakaryocytes. After labeling, megakaryocytes were analyzed by flow cytometry on a BD Cytoflex. In some experiments, megakaryocytes were also visually examined by confocal microscopy. To examine uptake of S. aureus and CPG ODN by megakaryocytes, megakaryocytes were incubated with 25 μg/mL of FITC-labeled Staphylococcus aureus (Wood Strain without protein A) or 40 μg/mL of 2395 ODN CPGs for 30 minutes after 24 hours after IFNα treatment. Megakaryocytes were then washed three times with PBS and labeled with MWReg30 before analyzing for uptake by flow cytometry. In additional experiments, vehicle or IFN-stimulated megakaryocytes were treated with 50 μg total of Alexa-Fluor labeled transferrin in the presence of a GPIb antibody (Emfret, 647). After 30 minutes, the cells were washed and imaged by confocal microscopy.

*Lipid Raft Analysis*

For lipid raft analysis, 1 x 10^7 pZIP empty or pZIP overexpressing IFITM3 MEG-01, 1 x 10^7 $CD34^+$-derived megakaryocytes treated with vehicle or 1000 U/mL IFN for 24 hours, or 4 x 10^9 healthy donor or septic platelets were used. Cells were pelleted and lysed with 2 mL of 1% Triton X-100 lysis buffer and incubated on ice for 30 minutes. After 30 minutes, 2 mL of lysed cells were mixed with 2 mL of 80% (w/v) sucrose in MES-buffer (25 mM Mes, pH6.5, 150 mM NaCl), resulting in a 40% (w/v) solution. This solution was then placed in 14 mL ultracentrifuge tube. A 30% (w/v, 4 mL total) sucrose solution in MBS was then layered on top of the platelet solution followed by a 5% (w/v, 4 mL total) sucrose solution in MBS. The resulting gradient was roughly 12 mL total. The gradient was then centrifuged at 200,000 x g for 18 hours at 4°C. After 18 hours, roughly 11, 1.1 mL fractions were removed. The fractions were then treated 0.6 N HClO4 to precipitate the protein and treated as described above. The samples were then probed by western blot analysis for IFITM3, clathrin, and $\alpha$IIb, and LAT.

*Aggregation*

Aggregation was measured using a lumi-aggregometer (Chrono-Log, Havertown, PA) at 37°C under stirred conditions as previously described(4, 7). Human and murine washed platelets ($2 \times 10^8$/mL 0.5ml) were stimulated with different agonists and the change in light transmission measured. In some experiments, 1, 10 or 25 $\mu$g/mL of human fibrinogen was added to examine the role of fibrinogen in mediating increased aggregation.

*Murine Model of Sepsis: Cecal Ligation and Puncture (CLP*

For experiments where *Ifitm3* mRNA was measured by qRT-PCR, bone marrow megakaryocytes were collected on day 1 and day 3 and platelets were collected by cardiac puncture on day 1, 2, 3, or 7 following CLP as previously described using C57BL/6J mice (Jackson Laboratory)(2, 3, 13). Untreated, age and sex-matched C57BL/6J mice were used as controls.

*Ferric Chloride Thrombosis Model.*

Mice were anesthetized by inhalation of 5% isoflurane and maintained under anesthesia by inhalation of 2% isoflurane. Buprenorphine was administered one hour before surgery. The right carotid artery was exposed and thrombosis was induced by topical application of a filter paper soaked in 10% $FeCl_3$ for 3 minutes. Excessive $FeCl_3$ was rinsed with PBS and blood flow was measured downstream of the application site with a laser Doppler flow probe for up to 20 minutes.

**Supplemental Table 1. Clinical Characteristics of Healthy Donors and Septic Patients**

| | Healthy Donors (n=23) | Septic Patients Patients (N=45) | p value |
|---|---|---|---|
| **Age (mean, ±SD)** | 56.5 (± 12.8) | 56.9 (± 16.7) | 0.92 |
| **Male (%)** | 48% | 58% | 0.45 |
| **Hispanic/Latino (%)** | 0% | 11.1% | 0.16 |
| **BMI (mean, ±SD)** | - | 33.0 (±12.5) | - |
| **Diabetes (%)** | 0% | 31.1% | 0.003 |
| **Hypertension (%)** | 0% | 53.3% | <0.0001 |
| **SOFA score (mean, ±SD)** | - | 7.8 (± 3.5) | |
| **Mechanical Ventilation (%)** | - | 20% | - |
| **Shock requiring Vasopressors (%)** | - | 46.7% | - |
| **90-day Survival (%)** | - | 93% | - |
| **Aspirin (%)** | 0% | 24.2% | 0.009 |
| **Platelet Count (mean, ±SD)*** | - | 197.0 (± 10.3) | - |
| **White Blood Count (mean, ±SD)**** | - | 15.9 (± 7.4) | - |

*Reference Range: 159-439 K/μL
**Reference Range: 4.3-11.3 K/μL

Supplemental Table 2

Please see associated excel file

Supplemental Table 3

Please see associated excel file

**Supplemental Table 4. Sources of Sepsis**

| | Patients (N=45) |
|---|---|
| **Pneumonia (%)** | 24% |
| **Urosepsis (%)** | 31% |
| **Skin and Soft Tissue Infection (%)** | 24% |
| **Other (%)** | 7% |
| **Unknown (%)** | 13% |
| | |

**Supplemental Table 5. Microorganism Associated with Sepsis**

| | Patients (N=45) |
|---|---|
| ***Escherichia coli* (%)** | 13% |
| ***Klebsiella pneumoniae* (%)** | 2% |
| ***Streptococcus* (%)** | 11% |
| ***Staphylococcus* (%)** | 7% |
| **Yeast (%)** | 4% |
| **Unknown* (%)** | 62% |
| | |

*no microorganism positively identified

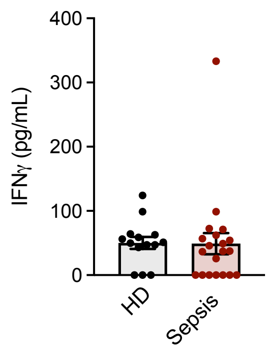


**Supplemental Figure 1. IFNγ levels are similar in healthy donors and non-viral sepsis patients.** IFNγ levels were measured in age, gender-matched healthy donors (HD, n=14) and non-viral sepsis patients (n=20) by ELISA. Statistical analysis used was a Mann-Whitney U test.

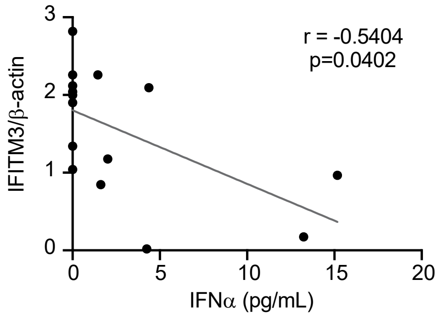


**Supplemental Figure 2. Plasma IFNα levels in sepsis patients negatively correlate with platelet IFITM3 expression.** Plasma IFNα levels and platelet IFITM3 protein expression (normalized to actin and square root transformed) were measured in non-viral sepsis patients upon study entry (n=15). The statistical analysis used was a Spearman correlation analysis.

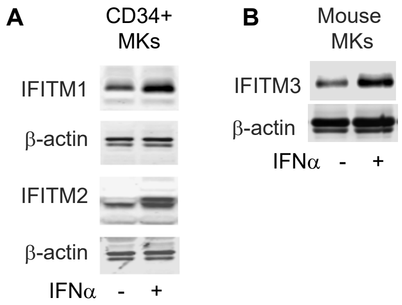


**Supplemental Figure 3. IFNα induces IFITM1 and IFITM2 in CD34⁺-derived megakaryocytes.** CD34⁺-derived megakaryocytes were stimulated with IFNα (1000 U/mL, final) or vehicle control on day 13. After 24 hours, megakaryocytes were lysed and probed form IFITM1, and IFITM2 expression by immunoblot. Actin was used as a loading control. A representative blot of IFITM1 and IFITM2 expression is shown (n=3). **B)** IFNα (1000 U/mL, final) induces Ifitm3 expression in murine bone marrow derived megakaryocytes (n=4). Actin was used as a loading control.

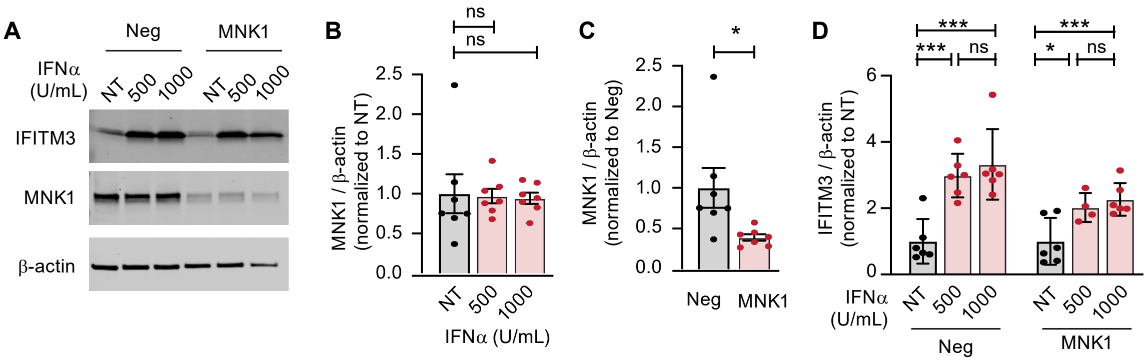


**Supplemental Figure 4. MNK1 deletion in CD34⁺-derived megakaryocytes does not alter IFITM3 expression.** CD34⁺ cells were transfected on day 5 of culture with negative (Neg) control or MNK1 crRNA (MNK1). Megakaryocytes were then left untreated (NT) or stimulated with IFNα (500 or 1000 U/mL, final) on day 13. After 24 hours, megakaryocytes were lysed and probed by immunoblot. Panel A shows a representative blot of MNK1 and IFITM3 expression (actin was used as a loading control) following IFNα stimulation in control (Neg) or MNK1 crRNA (MNK1) megakaryocytes**.** Panel B shows quantified MNK1 protein expression in control megakaryocytes following IFNα stimulation. Panel C shows quantified MNK1 protein expression in control (Neg) or MNK1 crRNA (MNK1) megakaryocytes. Panel D shows quantified IFITM3 protein expression following IFNα stimulation in negative (Neg) control or MNK1 crRNA (MNK1) megakaryocytes. The statistical analyses used were a Friedman test with a Dunn's multiple comparison test with values normalized to NT (**B**), a Wilcoxon test matched-pairs signed rank test with values normalized to Neg (**C**), and a mixed-effect analysis with a Sidak multiple comparison test with values normalized to NT (**D**) (n=5-7). *P ≤ 0.05. ***P ≤ 0.001.

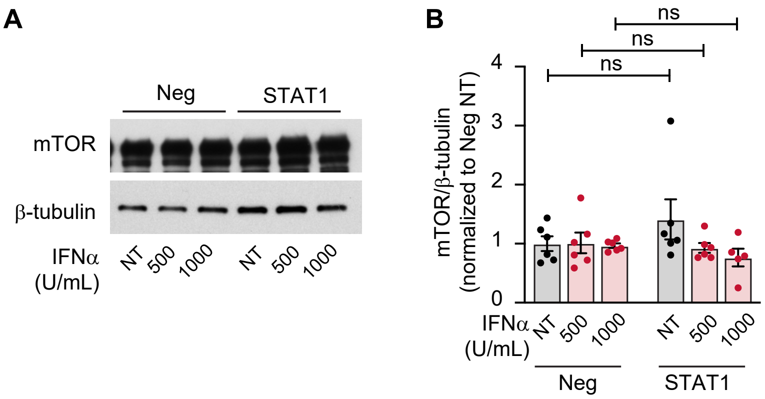


**Supplemental Figure 5. STAT1 deletion in CD34⁺-derived megakaryocytes does not alter mTOR expression. (A-B)** CD34⁺ cells were transfected on day 5 of culture with negative (Neg) control or STAT1 crRNA. Megakaryocytes were then stimulated with IFNα (500 or 1000 U/mL, final) or no treatment (NT) control on day 13. After 24 hours, megakaryocytes were lysed and probed by immunoblot. Tubulin was used as a loading control. (**A**) Representative blot and (**B**) quantification of mTOR expression (n=6-7). Statistical analysis used was a mixed-effect analysis with a Tukey's multiple comparison test with values normalized to Neg NT.

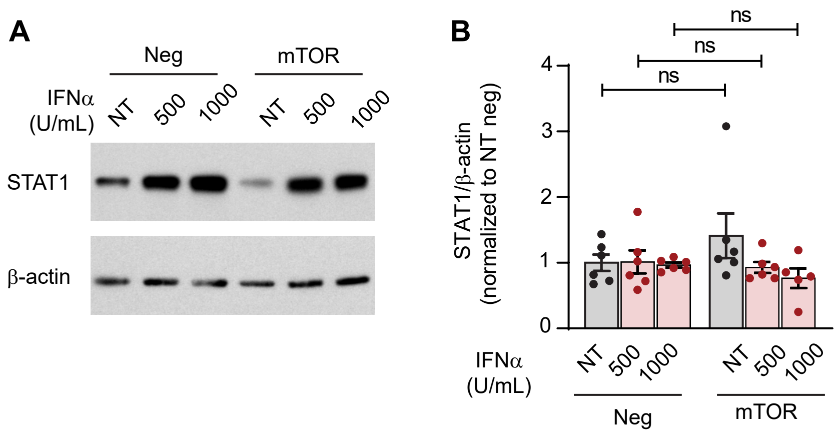


**Supplemental Figure 6. mTOR deletion in CD34⁺-derived megakaryocytes does not alter STAT1 expression.** CD34⁺ cells were transfected on day 5 of culture with negative (Neg) control or mTOR crRNA (mTOR). Megakaryocytes were then stimulated with IFNα (500 or 1000 U/mL, final) or vehicle control on day 13. After 24 hours, megakaryocytes were lysed and probed by immunoblot to determine if IFNα altered total STAT1 protein expression. Actin was used as a loading control. (**A**) Representative immunoblot and (**B**) quantification of STAT1 protein expression (n=6-7). The statistical analysis used was a mixed-effect analysis with a Sidak multiple comparison test with values normalized to Neg NT. The actin in Supplemental Figure 6A is the same actin in Figure 2E.

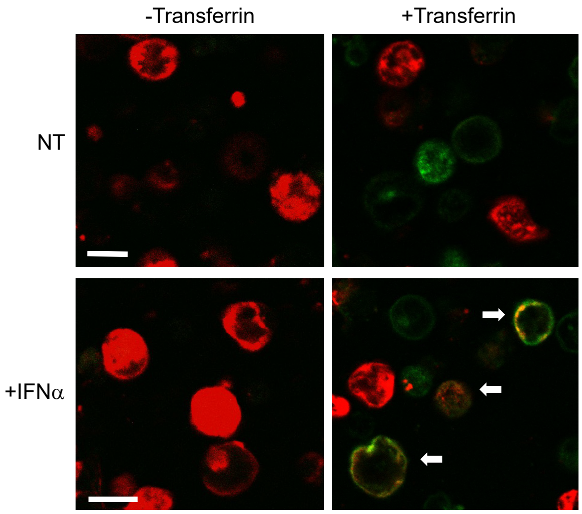


**Supplemental Figure 7. IFNα stimulation increases transferrin endocytosis in murine megakaryocytes.** Bone marrow-derived megakaryocytes were cultured for four days before stimulation with vehicle or murine IFNα (1000 U/mL, final) for 24 hours. After 24 hours, megakaryocytes were isolated by density gradient and incubated with vehicle or Alexa-fluor 488 transferrin (50 mg, total) for 30 minutes. Megakaryocytes were then washed three times and stained with Dylight649 GPIbα to identify megakaryocytes. Transferrin positive megakaryocytes were identified by confocal microscopy (white arrows). The images are representative of three independent experiments. Scale bar is 20 µm.

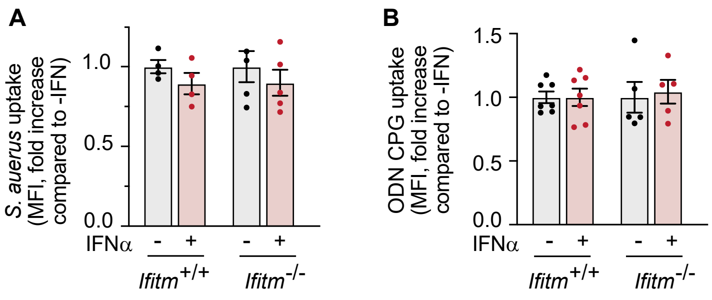


**Supplemental Figure 8. Endocytosis of *S. aureus* and CPG ODN is independent of IFITM expression.** Murine bone marrow megakaryocytes were isolated from *Ifitm*$^{+/+}$ and *Ifitm*$^{-/-}$ mice and cultured for four days before stimulation with IFNα (1000 U/mL, final) for 24 hours. After 24 hours, 25 μg/mL of FITC-labeled Staphylococcus aureus (Wood Strain without protein A) (**A,** n=4-5/group) or 40 μg/mL of 2395 ODN CPGs (**B**, n=5-7/group) was added to the cells. After a 30-minute incubation, megakaryocytes were washed three times with PBS and stained with CD41 to identify mature megakaryocytes. CD41 and *S. aureus* or CD41 and 2395 ODN CPG positive megakaryocytes were then measured by flow cytometry. Statistical analysis used was a mixed-effect analysis with a Sidak multiple comparison test with values normalized to -IFNα in each group.

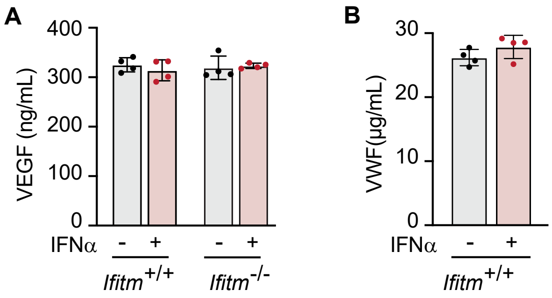


**Supplemental Figure 9. Platelet VEGF and VWF levels are independent of IFITM expression**. *Ifitm*$^{+/+}$ and *Ifitm*$^{-/-}$ mice were injected with vehicle or murine IFNα (25,000 U per injection) once daily for three consecutive days. Platelets were then isolated and total **(A**, n=4/group**)** VEGF levels and (**B**, n=4/group) vWF levels measured by ELISA. Statistical analyses used were (**A**) a one-way ANOVA with a Sidak multiple comparison test and (**B**) an unpaired t-test.

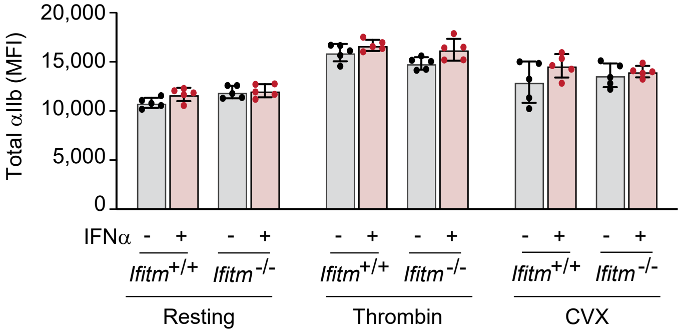


**Supplemental Figure 10. Surface αIIb levels remain unchanged after IFNα.** *Ifitm*$^{+/+}$ and *Ifitm*$^{-/-}$ mice were injected with or without murine IFNα (25,000 U per injection) once daily for three consecutive days. Platelets were then isolated and stained for αIIb. Surface αIIb levels were then measured by flow cytometry under resting conditions and after activation with thrombin (0.2 U/mL, final) or convulxin (CVX, 100 ng/mL, final). Statistical analysis used was a two-way ANOVA with a Dunnett's multiple comparison test. n=5 per group.

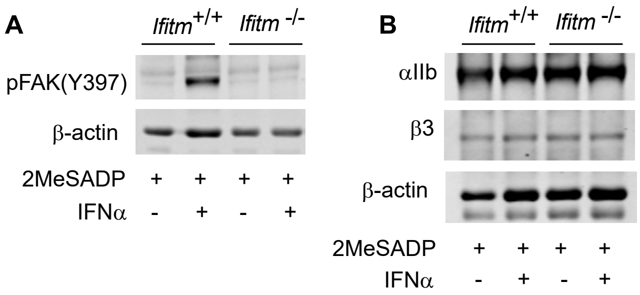


**Supplemental Figure 11. IFNα increases fibrinogen endocytosis while total αIIbβ3 levels remain unchanged.** (**A**) Washed platelets were activated with 2MesADP and downstream activation of integrin β3 was measured by probing for pFAK. β-actin was used as a loading control. The immunoblot is a representative example from an n= 3. **(B)** *Ifitm* $^{+/+}$ and *Ifitm* $^{-/-}$ mice were injected with vehicle or murine IFNα (25,000 U per injection) for three consecutive days. On day four, washed platelets were isolated and αIIb and β3 were examined by immunoblot. β-actin was used as a loading control. The immunoblot is a representative example from an n= 3.

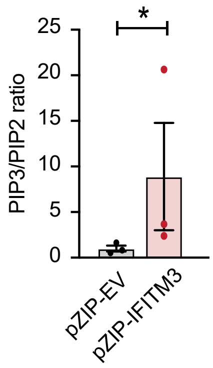


**Supplemental Figure 12. IFITM3 increase PIP3 levels in megakaryocytes.** pZIP-EV control or pZIP-IFITM3 overexpressing MEG-01s were stimulated with PMA (100 nM, final) for 24 hours. After 24 hours, MEG-01s were harvested (>45 million per condition). The cells were lysed and split to measure PIP3 and PIP2 levels using a PIP3 Mass ELISA or a PI(4,5)P2 Mass ELISA kit. Statistical analysis used was a Mann-Whitney U one-tailed test. n=3 per group. $^*P \leq 0.05$.

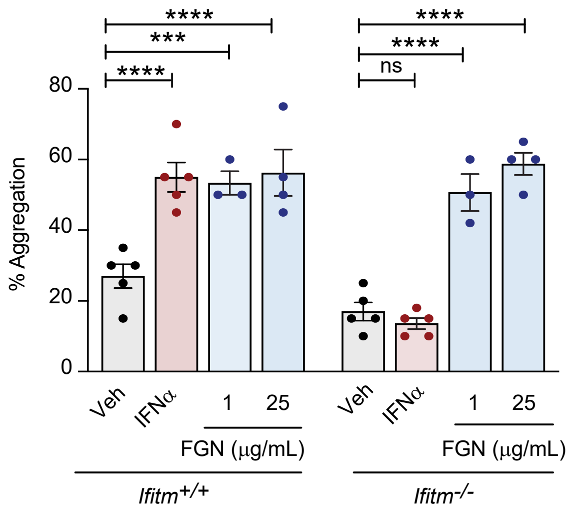


**Supplemental Figure 13. The addition of exogenous fibrinogen rescues IFITM-dependent platelet aggregation**. Washed platelets were isolated from *Ifitm*$^{+/+}$ and *Ifitm*$^{-/-}$ mice treated with vehicle (Veh) or murine IFNα (25,000 U per injection) for three consecutive days. Separately, platelets were isolated from unstimulated *Ifitm*$^{+/+}$ and *Ifitm*$^{-/-}$ mice, and then 1 μg/mL or 25 μg/mL of exogenous fibrinogen (FGN) added to isolated platelets. Maximal aggregation was then measured in response to 2MesADP (10 nM, final). n=3-5 per group. Statistical analysis used was a one-way ANOVA with a Sidak's multiple comparisons test. ***P ≤ 0.001. ****P ≤ 0.0001.

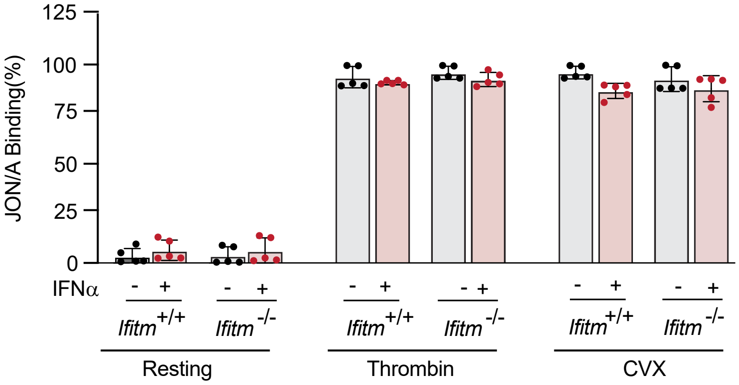


**Supplemental Figure 14. Integrin αIIb activation is not altered by IFNα or IFITM.** Integrin activation as measured by JON/A binding was measured in washed platelets from *Ifitm*$^{+/+}$ and *Ifitm*$^{-/-}$ mice treated with vehicle or murine IFNα (25,000 U per injection) for three consecutive days in response to thrombin (0.2 U/mL, final) or convulxin (CVX, 100 ng/mL, final). n=5 per group. Statistical analysis used was a two-way ANOVA with a Tukey's multiple comparisons test.

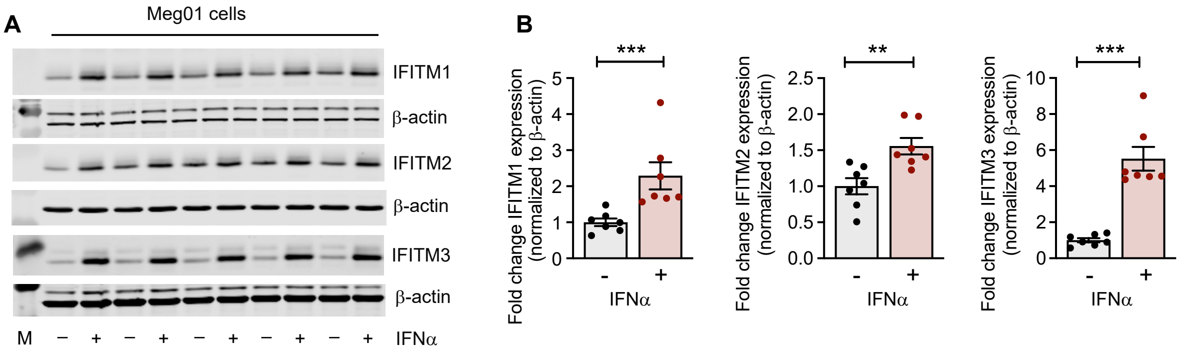


**Supplemental Figure 15. IFNα induces IFITM3 expression in MEG-01s**. (**A-B**) MEG-01s were stimulated PMA (100 nM, final) to induce differentiation. After 24 hours, MEG-01s were stimulated with IFNα (1000 U/mL, final) and IFITM1, IFITM2, and IFITM3 expression was measured by immunoblot. Actin was used as a loading control. The immunoblot is representative from an n= 7. Statistical analysis used was a Mann-Whitney U test. $^{**}P \leq 0.01$ and $^{***}P \leq 0.001$.

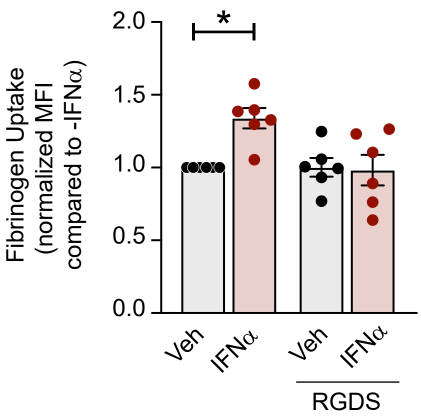


**Supplemental Figure 16. RGDS peptide inhibits IFITM3 mediated endocytosis**. MEG-01 megakaryocytes were stimulated with PMA (100 nM, final) to induced differentiation. After 24 hours, MEG-01s were stimulated with Vehicle (Veh) or IFNα (1000 U/mL, final) and 24 hours later incubated with 10mg Alexa-Fluor 546 labeled fibrinogen for 30 minutes in the presence or absence of Cyclo-RGDfK (1 μM). The MEG-01s were then washed and labeled with a CD41 antibody and CD41 positive MEG-01s were measured for fibrinogen endocytosis by flow cytometry (n=6 per group). Statistical analysis used was a Kruskal-Wallis test with a Dunn's Multiple comparison test compared to minus IFNα. *P ≤ 0.05.

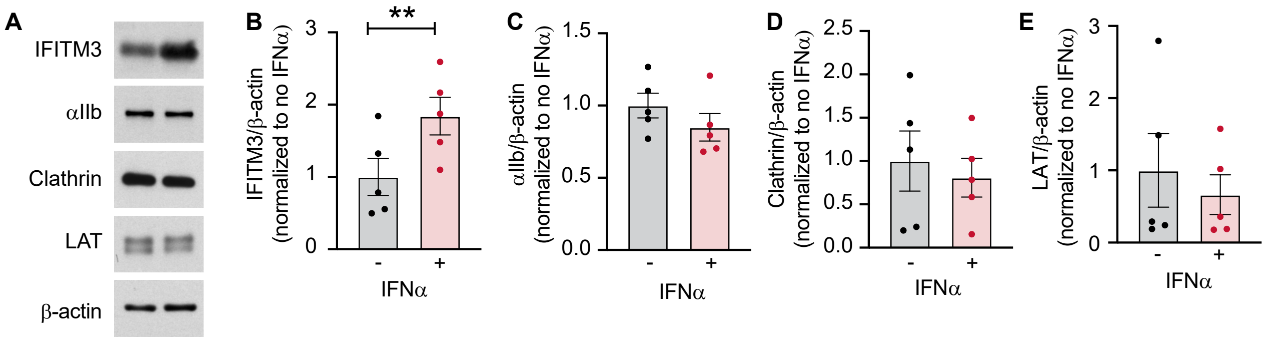


**Supplemental Figure 17. IFNα stimulation does not alter total αIIb, clathrin, and LAT expression in CD34⁺-derived megakaryocytes.** CD34⁺-derived megakaryocytes were stimulated with vehicle or IFNα (1000 U/mL, final). (**A-E**) IFITM3 (**B**), αIIb (**C**), clathrin (**D**), LAT (**E**), a lipid raft specific marker, and actin, a loading control, were probed by immunoblot. Statistical analysis used was a paired t-test. **P ≤ 0.01. n= 5 per group.

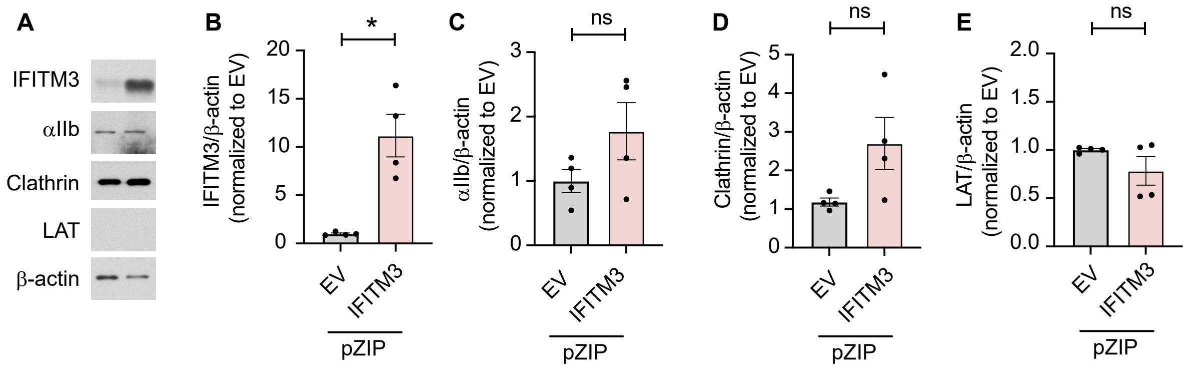


**Supplemental Figure 18. IFITM3 overexpression does not alter total αIIb, clathrin, and LAT expression in MEG-01s.** MEG-01s were transfected with an empty vector control (pZIP+EV) or an engineered vector containing IFITM3 (pZIP-IFITM3) were lysed. (**A-E**) IFITM3 (**B**), αIIb (**C**), clathrin (**D**), LAT (**E**), and β-actin were probed by immunoblot. Statistical analysis used was a paired tt -test (**B, C, D**) or Wilcoxon Rank Sum test (**E**). *P ≤ 0.05. n=4 per group.

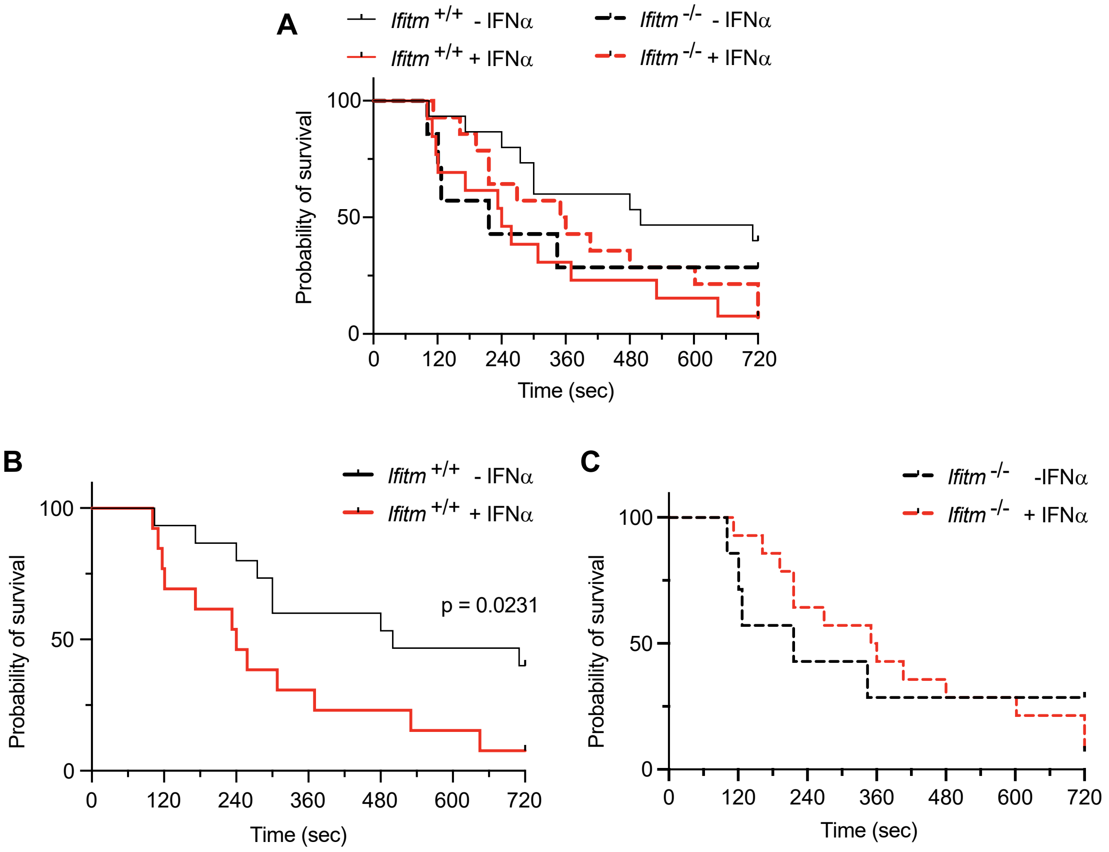


**Supplemental Figure 19. IFITMs regulate pulmonary thrombosis upon IFNα stimulation. (A-C)** *Ifitm*$^{+/+}$ and *Ifitm*$^{-/-}$ mice were injected with vehicle or murine IFNα (25,000 U per injection) once daily for three consecutive days. Mice were then subjected to a collagen/epinephrine pulmonary microvascular thrombosis model by i.v. injection of collagen/epinephrine. The time to cessation of breathing was measured in seconds (n=12-17 per group). Experiments were stopped after 720 seconds. Statistical analysis used was Mantel-Cox Log-Rank test.

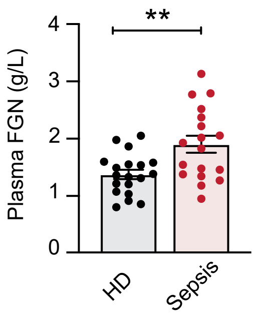


**Supplemental Figure 20. Plasma fibrinogen levels are increased during sepsis**. Plasma was isolated from healthy donors (HD) or non-viral sepsis patients and plasma fibrinogen levels measured using an ELISA. Statistical analysis used was an unpaired t-test. **P ≤ 0.01. n=18-19 per group.

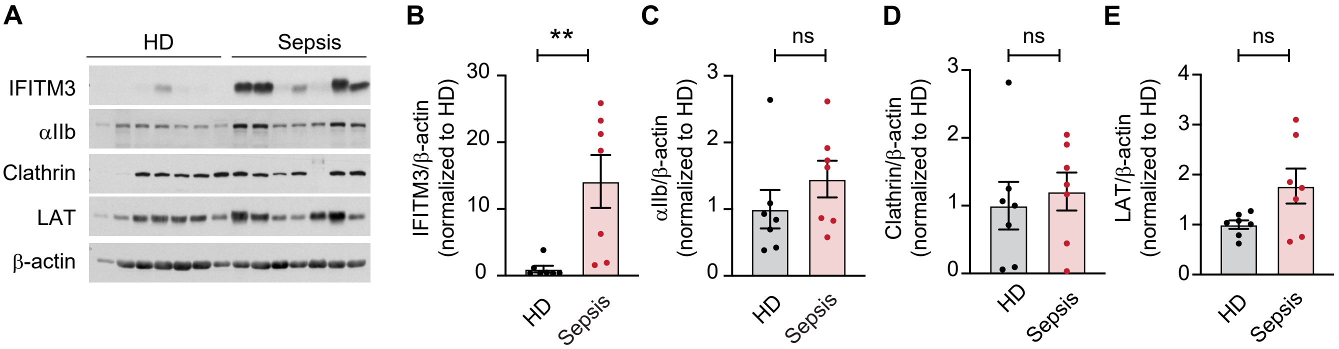


**Supplemental Figure 21. Total levels of αIIb, clathrin, and LAT protein are not significantly altered in sepsis patients.** Platelets isolated from healthy donors or non-viral sepsis patients were probed for IFITM3 (quantified in panel **B**), αIIb (quantified in panel **C**), clathrin (quantified in panel **D**), and LAT (quantified in panel **E**) protein (normalized to β-actin loading control). Panel **A** shows a representative immunoblot. Statistical analysis used was an unpaired-test (Panels **B, C, D**) or a Mann-Whitney test (Panel **E**). **P ≤ 0.01. n=7 per group.

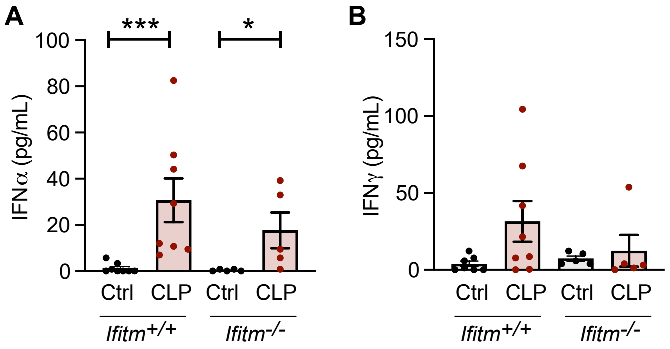


**Supplemental Figure 22. IFNα are elevated in septic mice compared to sham mice while IFNγ levels are similar.** Sepsis was induced in *Ifitm*$^{+/+}$ and *Ifitm*$^{-/-}$ mice by CLP and plasma was isolated 24 hours later. IFNα (**A**) and IFNγ (**B**) levels were measured in sham and CLP mice by ELISA (n=5-7 per group). Statistical analysis used was a Kruskal-Wallis test with a Dunn's Multiple comparison test. *P ≤ 0.05 and ***P ≤ 0.001.

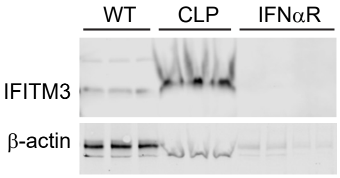


**Supplemental Figure 23 Anti-IFNαR treatment reduces platelet IFITM3 expression after CLP.** Sepsis was induced in *Ifitm*$^{+/+}$ mice by CLP. One hour before CLP and six hours after CLP, mice were treated with 1 mg (total, i.p.) of an anti-IFNαR1 antibody or control IgG. Platelets were isolated at day 3 and platelet IFITM3 expression measured by immunoblot. n=3-4.

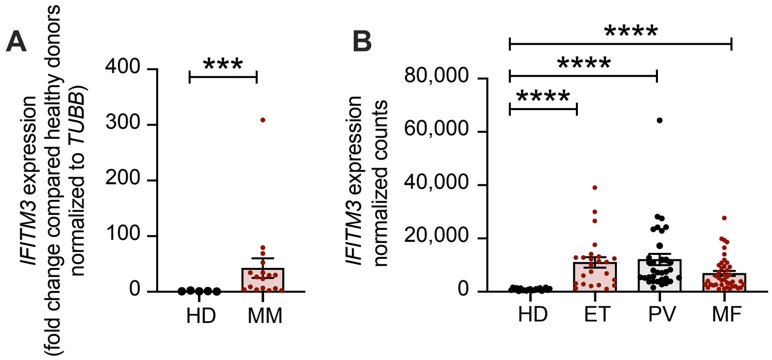


**Supplemental Figure 24. IFITM3 is increased in patients with myeloproliferative disorders and multiple myeloma.** (A) Platelets from healthy donors (HD) or patients with multiple myeloma (MM, n=17) were isolated and IFITM3 expression was measured and normalized to Tubulin (TUBB). HD were from a subset of donors recruited from sepsis studies (n=6). Statistical analysis used was a Mann-Whitney U Test. ***P ≤ 0.001. (B) Platelets from healthy donors (HD, n=21) or patients with myeloproliferative disorders were isolated and IFITM3 expression was measured by RNA-seq analysis. Data are represented by normalized counts. Myeloproliferative disorders were divided into subclassification: Essential Thrombocythemia (ET, n=24), Polycythemia Vera (PV, n=33), and Myelofibrosis (MF, n=42). Statistical analysis used was a Kruskal-Wallis test with a Dunn's Multiple comparison test. ****P ≤ 0.0001.

## References


1. Eustes AS, Campbell RA, Middleton EA, Tolley ND, Manne BK, Montenont E, et al. Heparanase expression and activity are increased in platelets during clinical sepsis. *J Thromb Haemost.* 2021;19(5):1319-30.
2. Guo L, Shen S, Rowley JW, Tolley ND, Jia W, Manne BK, et al. Platelet MHC class I mediates CD8+ T-cell suppression during sepsis. *Blood.* 2021;138(5):401-16.
3. Middleton EA, Rowley JW, Campbell RA, Grissom CK, Brown SM, Beesley SJ, et al. Sepsis alters the transcriptional and translational landscape of human and murine platelets. *Blood.* 2019;134(12):911-23.
4. Manne BK, Denorme F, Middleton EA, Portier I, Rowley JW, Stubben C, et al. Platelet gene expression and function in patients with COVID-19. *Blood.* 2020;136(11):1317-29.
5. Campbell RA, Franks Z, Bhatnagar A, Rowley JW, Manne BK, Supiano MA, et al. Granzyme A in Human Platelets Regulates the Synthesis of Proinflammatory Cytokines by Monocytes in Aging. *J Immunol.* 2018;200(1):295-304.
6. Kraemer BF, Campbell RA, Schwertz H, Cody MJ, Franks Z, Tolley ND, et al. Novel anti-bacterial activities of beta-defensin 1 in human platelets: suppression of pathogen growth and signaling of neutrophil extracellular trap formation. *PLoS Pathog.* 2011;7(11):e1002355.
7. Manne BK, Munzer P, Badolia R, Walker-Allgaier B, Campbell RA, Middleton E, et al. PDK1 governs thromboxane generation and thrombosis in platelets by regulating activation of Raf1 in the MAPK pathway. *J Thromb Haemost.* 2018;16(6):1211-25.
8. Davizon-Castillo P, McMahon B, Aguila S, Bark D, Ashworth K, Allawzi A, et al. TNF-alpha-driven inflammation and mitochondrial dysfunction define the platelet hyperreactivity of aging. *Blood.* 2019;134(9):727-40.
9. Campbell RA, Schwertz H, Hottz ED, Rowley JW, Manne BK, Washington AV, et al. Human megakaryocytes possess intrinsic antiviral immunity through regulated induction of IFITM3. *Blood.* 2019;133(19):2013-26.
10. Montenont E, Bhatlekar S, Jacob S, Kosaka Y, Manne BK, Lee O, et al. CRISPR-edited megakaryocytes for rapid screening of platelet gene functions. *Blood Adv.* 2021;5(9):2362-74.
11. Schwertz H, Rowley JW, Portier I, Middleton EA, Tolley ND, Campbell RA, et al. Human platelets display dysregulated sepsis-associated autophagy, induced by altered LC3 protein-protein interaction of the Vici-protein EPG5. *Autophagy.* 2022;18(7):1534-50.
12. Lee J, Robinson ME, Ma N, Artadji D, Ahmed MA, Xiao G, et al. IFITM3 functions as a PIP3 scaffold to amplify PI3K signalling in B cells. *Nature.* 2020;588(7838):491-7.
13. Yost CC, Schwertz H, Cody MJ, Wallace JA, Campbell RA, Vieira-de-Abreu A, et al. Neonatal NET-inhibitory factor and related peptides inhibit neutrophil extracellular trap formation. *J Clin Invest.* 2016;126(10):3783-98.
